# Transgenerational Imprints of Sequential Herbivory on Soybean Physiology and Fitness Traits

**DOI:** 10.1002/pei3.70070

**Published:** 2025-07-04

**Authors:** Insha Shafi, Rupesh Kariyat

**Affiliations:** ^1^ Department of Entomology and Plant Pathology University of Arkansas Fayetteville Arkansas USA

**Keywords:** fall armyworm, fitness traits, priming, soybean looper, transgenerational plasticity

## Abstract

Sequential herbivory leads to a multitude of effects on plants, influencing processes like growth, physiology, defense, and fitness. However, whether the sequential herbivore attack can elicit any transgenerational consequences is poorly examined. In this study, we show evidence of transgenerational impacts of sequential herbivory by two chewing herbivores, fall armyworm ( *Spodoptera frugiperda* , FAW) and soybean looper ( *Chrysodeixis includens* , SL) on soybean ( *Glycine max* ) progeny. Seeds from the parents subjected to different herbivore sequences (SL‐FAW: initial attack by SL followed by FAW and FAW‐SL: FAW followed by SL) were used for the transgenerational experiment. Our main hypothesis was that parental sequential herbivory would exert transgenerational effects on progeny traits leading to potential trade‐offs. Also, herbivore identity and sequence of attack would critically influence these outcomes. A comprehensive investigation of early growth, physiological performance, physical defense (trichomes) and fitness of the progeny was then carried out. Our results revealed that early growth traits like germination, root morphology, and nitrogen/protein content were similar among maternal treatments; however, significant transgenerational impacts of the sequential attack of SL‐FAW were observed in physiological and fitness traits. Notably, no transgenerational effects were observed in physical defenses (trichome density), suggesting an investment in physiology and fitness traits over physical defenses. Collectively, the results confirm that the sequence and identity of herbivore attack in the parental generation can critically shape transgenerational plant responses in soybean, providing novel insights on crop performance under multiple biotic stresses.

## 1 Introduction

To optimize fitness, plants can modulate the phenotypic traits of their progeny in response to environmental stressors experienced by the parental generation (Puijalon et al. 2008; Kellenberger et al. 2018). There is substantial evidence for this phenomenon known as “transgenerational plasticity,” that what was considered earlier as environmental noise (Agrawal 2001; Holeski 2007; Rasmann et al. 2012; Colicchio 2017). Independent of genetic changes, transgenerational phenotypic plasticity exhibits altered phenotypes in progeny in response to environmental cues perceived by parents (Agrawal et al. 1999; Galloway and Etterson 2007; Tariel et al. 2020). These transgenerational responses are mainly shaped through two different phenomena—maternal effects and epigenetic modifications—each with distinct impacts on offspring phenotype (Roach and Wulff 1987; Herman and Sultan 2011). Although maternal effects such as nutrient provisioning to progeny have been well understood, it has been recently reported how epigenetic effects can be heritable through processes like DNA methylation (Richards 2011), histone modifications (Rapp and Wendel 2005), small RNA influencing gene expression or even homologous recombination (Boyko and Kovalchuk 2011). Together these phenomena can have transgenerational impacts. Under biotic or abiotic stresses, these mechanisms equip plants to adjust to immediate environmental challenges and to transmit adaptive information across generations.

A growing body of research has documented transgenerational effects of biotic stress (e.g., insect herbivory) across different crops (Agrawal 2001, 2002; Rasmann et al. 2012; Holeski et al. 2012; Fu et al. 2023). The progeny from herbivore‐damaged plants can become more resistant to herbivore infestations, a process called defense priming, which can lead to enhanced chemical (Rasmann et al. 2012; Ballhorn et al. 2016) or physical defenses (Holeski 2007; Nihranz et al. 2022). Recent investigations by de Souza and Peñaflor (2024) on green peach aphid ( *Myzus persicae* ) herbivory of bell pepper plants ( *Capsicum annuum* ) reported induction of resistance in aphid‐infested transgenerational plants; this coincided with elevated chemical and indirect defenses, albeit at the cost of early growth. Herbivore damage in *Arabidopsis* and tomato ( *Solanum lycopersicum* ) plants resulted in enhanced jasmonate responsiveness to damage in the next generation (Rasmann et al. 2012). Similar transgenerational priming on trichome induction in progeny of yellow monkeyflower ( *Mimulus guttatus* ) plants due to parental herbivory was observed by Holeski (2007). Maternal herbivory by *Manduca sexta* caterpillars on horsenettle plants, *Solanum carolinense* plants resulted in enhanced physical and chemical defenses in transgenerational plants, with reduced growth of caterpillars (Nihranz et al. 2022). Ballhorn et al. (2016) reported elevated cyanogenesis in progeny of wild lima bean ( *Phaseolus lunatus* ) exposed to herbivore *Gynandrobrotica guerreroensis*. However, the effect vanished within 4 weeks, highlighting the transient nature of transgenerationally induced defenses. Conversely, reverse transgenerational priming effects were reported by Agrawal (2002) in wild radish ( *Raphanus raphanistrum* ), wherein maternal jasmonate led to reduced defense responses to caterpillars in the progeny plants. This variability in transgenerational priming necessitates the need to understand how a combination of stressors can shape transgenerational responses in plants, especially in agroecosystems where seed storage and multi‐generational farming practices are more common. Most of the studies investigating transgenerational plant responses to herbivory have focused on the effect of single stressors. However, in agroecosystems, plants are attacked by herbivores either singly or sequentially (Kundu et al. 2023). To our knowledge, no study has yet explored the transgenerational effects of sequential herbivory on high acreage crops like soybean.

We recently assessed the effects of sequential herbivory in soybean ( *Glycine max* ) by two important chewing herbivores: Fall armyworm ( *Spodoptera frugiperda* , FAW) and Soybean looper ( *Chrysodeixis includens* , SL). SL is considered a major defoliator of soybean, whereas FAW is a minor but highly polyphagous pest, able to feed on plants from taxonomically diverse families (Souza et al. 2021; Nagoshi et al. 2025). We challenged soybeans with these herbivores for 96 h (fourth instar caterpillar per plant) in different sequential combinations, the important ones being FAW‐SL (FAW followed by SL) and SL‐FAW (SL followed by FAW), representing ecologically relevant attack sequences. The key findings from our earlier study demonstrated that sequential herbivory modulated morpho‐physiological traits of maternal soybean; however, overall fitness and yield remained unaltered (unpublished data). Although the transgenerational impacts of single herbivory stress have been reported recently on wild soybean ( *Glycine soja* ), resulting in earlier pod maturation and larger seeds compared to control plants (Adachi‐Fukunaga et al. 2022). However, a critical question that remains unanswered is whether sequential herbivory leaves any transgenerational imprints.

In our current study, we addressed this knowledge gap by investigating the transgenerational impacts of sequential herbivory by FAW and SL on soybean offspring traits, using two cultivars, *Blackhawk* and *Magellan*, with a focus on growth, nutrition, defense, physiology, and overall fitness. Our specific goals were to determine whether sequential herbivory of FAW and SL in maternal plants affects (1) soybean growth, including germination percentage, early root morphology, and cotyledon nitrogen/protein content (2) physical defense traits, specifically trichome density; (3) physiological traits such as net photosynthetic rate (μmmol CO_2_ m^−2^ s^−1^), stomatal conductance (mol m^−2^ s^−1^), transpiration rate (mmol H_2_O m^−2^ s^−1^), and intercellular CO_2_ concentration (μmol CO_2_ mol^−1^), and (4) yield and fitness traits (flower count, pod and seed traits). We hypothesized that parental sequential herbivory would have a transgenerational impact on traits associated with offspring growth, defense, physiology, and reproductive success. Specifically, we also predicted that progeny of SL‐FAW treatment plants would be strongly primed compared to FAW‐SL progeny, owing to the host specificity of SL for soybeans.

## 2 Materials and Methods

### 2.1 Plant Materials

**FIGURE 1:**
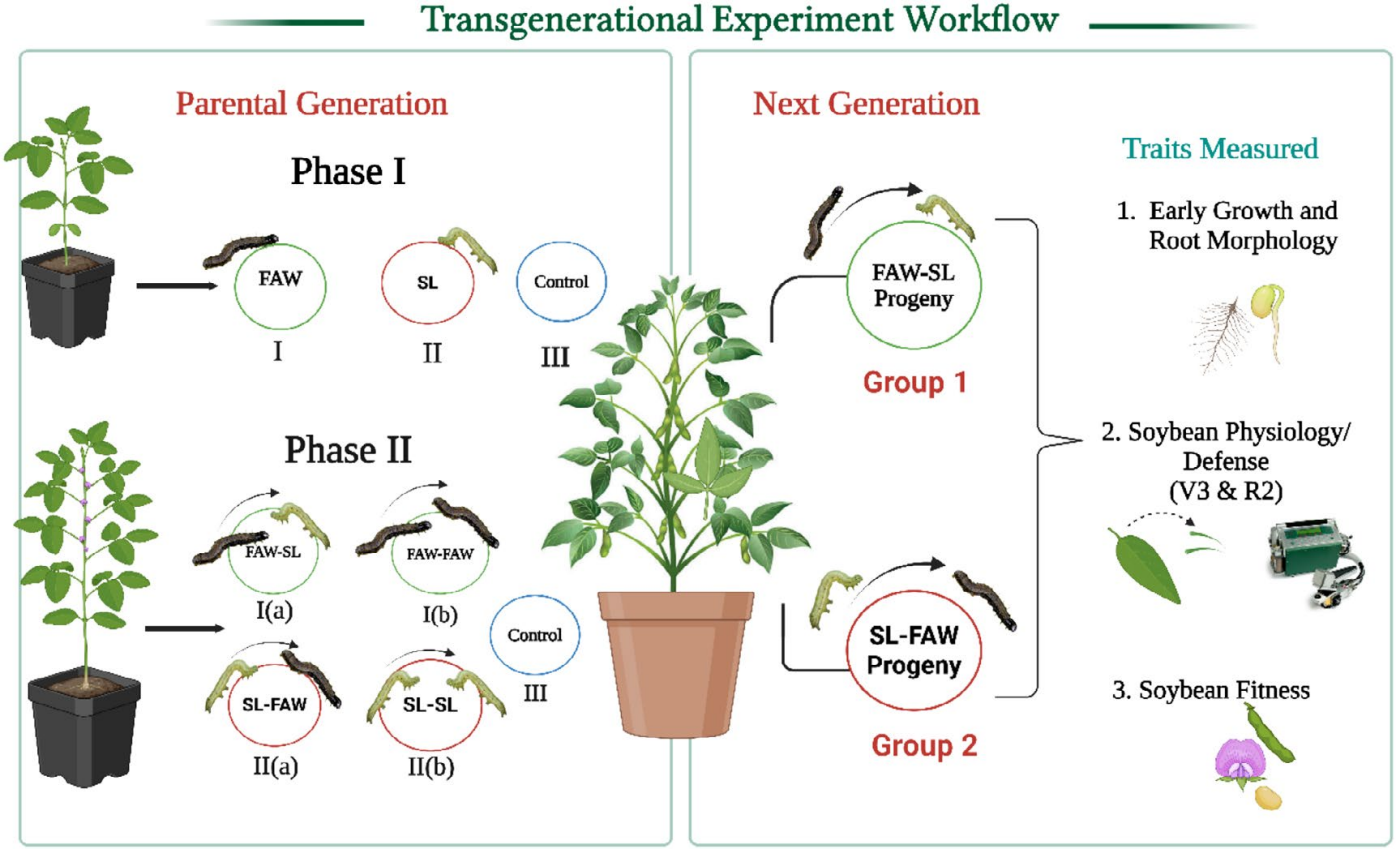
Schematic representation of the transgenerational effects of sequential herbivory on two soybean cultivars. In the parental generation, the plants were exposed to attacks from fall armyworm (FAW) and soybean looper (SL) in phase I (at vegetative stage: V3) and in phase II, plants at the reproductive stage (R2) were subjected to FAW and SL attack in a sequential manner, resulting in four sub‐groups: (i) FAW‐FAW = sequential attack by FAW, (ii) FAW‐SL = Initial attacker is FAW and SL is the second attacker, (iii) SL‐SL (sequential attack by SL), and (iv) SL‐FAW (Initial attacker is SL and FAW is the second attacker), and a control group (no herbivory). The treatment combinations from SL‐FAW and FAW‐SL were planted again in the next generation for assessing transgenerational effects. Early growth traits, physiological traits, and fitness traits were assessed.

The soybean cultivars (Blackhawk and Magellan) used during the sequential herbivory experiment (Figure 1) served as the source of seed material for the transgenerational experiment (unpublished data). In the parental generation, plants were exposed to sequential herbivore attacks by FAW and SL in different combinations. The seeds collected from two treatment combinations: FAW‐SL (FAW: initial attack; SL: sequential attack) and SL‐FAW (SL: initial attack; FAW: sequential attack) were used for the transgenerational studies. The plants were grown in Pro‐Mix BX General Purpose Growing Mix (Premier Tech Horticulture, Lawrenceville, GA, USA), a peat‐based substrate, in a greenhouse at the Milo J. Shult Agricultural Research & Extension Center (SAREC), Arkansas Agricultural Experiment Station of University of Arkansas (under controlled conditions of 70% RH, a 16:8 h light–dark cycle, and a temperature range of 28°C–30°C; Gautam et al. 2024).

### 2.2 Sequential Herbivory in Parental Generation

The plants were subjected to two phases of herbivory in the parental generation. During the initial herbivory phase, plants at the V3 stage (three fully formed trifoliate leaves) were divided into three groups: (i) FAW‐infested, (ii) SL‐infested, and (iii) control group (no herbivory). These plants were allowed to grow till they reached the R2 stage (full bloom stage). At the R2 stage, the previously infested plants were again challenged by herbivory, either FAW or SL, resulting in different treatment combinations: (1) Dual herbivore attack: (i) FAW‐FAW; (ii) SL‐SL; (2) Altered herbivore sequence: (i) FAW‐SL; (ii) SL‐FAW; and (3) Control group (no herbivory). For our transgenerational experiment, we planted seeds from all these combinations but focused our analysis specifically on the progeny from the altered herbivory group (FAW‐SL and SL‐FAW). The reason for this selection was two‐fold: logistical constraints that limited our ability to do comprehensive evaluations, but more importantly, to isolate the effects of herbivore sequence and order of attack across generations while minimizing confounding factors. The conditions for growing the transgenerational studies were similar to our parental germination with the motive to maintain environmental consistency between experiments.

### 2.3 Germination Percentage in Transgenerational Plants

The germination rate of the plants (FAW‐SL and SL‐FAW; *n* = 100 per treatment) was measured to assess the transgenerational effects of sequential herbivory. The germination data was recorded for a period of 5 days, with the emergence of cotyledons out of the growing media used as the criterion for germination.

### 2.4 Root Morphology

A comprehensive examination of root traits was carried out using roots of 2‐week‐old seedlings from FAW‐SL and SL‐FAW treatment groups (*n* = 6 per treatment). Roots were washed to remove soil and imaged using the WinRHIZO root scanning system (Regent Instruments Inc., Quebec, Canada) (Tripathi et al. 2021; Gautam et al. 2024). The traits measured were total root length (cm), fine root length (roots ≤ 2 mm diameter), root volume (cm^3^), fine root volume (cm^3^), surface area (cm^2^) number of root tips, number of root forks, and number of crossings.

### 2.5 Nutrient Analysis of Cotyledons

The cotyledons from 2‐week‐old seedlings of the two treatment progeny plants (FAW‐SL and SL‐FAW) were collected in zip‐lock bags on dry ice and then stored in a freezer (−80°C) until the samples were ready for processing. Samples were crushed to a fine powder in a mortar and pestle using liquid nitrogen. Nitrogen and protein percentages (*n* = 4 each treatment) were analyzed using the Dumas Combustion method at the Central Analytical Laboratory, University of Arkansas, following the AOAC 968.06‐1969 protocol for crude protein determination in plant and feed materials. A Fisons NA2000 Carbon Nitrogen Analyzer was used for this purpose, which combusts the dried, finely ground samples at high temperatures in the presence of pure oxygen (University of Arkansas System Division of Agriculture, 2024; https://cal.uada.edu/nutrition‐services/).

### 2.6 Plant Physiological Traits at Two Phenological Stages

Plant physiological traits like net photosynthetic rate (μmmol CO_2_ m^−2^ s^−1^), stomatal conductance (mol m^−2^ s^−1^), transpiration rate (mmol H_2_O m^−2^ s^−1^), and intercellular CO_2_ concentration (μmol CO_2_ mol^−1^) were measured from progeny using LI‐COR portable photosynthetic systems (Model LI‐6400/XT) (LI‐COR Biosciences, Lincoln, NE, USA). Six plants were randomly selected, and the topmost fully opened leaf from both treatments (imposed in parental generation) from each cultivar was used for recording these measurements. Measurements were taken on sunny days between 9:00 to 12:00 h, under the following standardized chamber conditions: photosynthetically active photon flux (1500 μmol m^−2^ s^−1^) environment CO_2_ concentration (400 mol m^−2^ s^−1^) and chamber humidity (45%–55%) (Bruno et al. 2009). The data was collected at two different phenological stages of the plants (V3 and R2), consistent with the parental generation.

### 2.7 Physical Defense (Trichome Density) at Two Phenological Stages

Topmost fully mature leaves of transgenerational plants at V3 and R2 stages under FAW‐SL and SL‐FAW treatments were used to estimate the trichome density. Leaf discs from 10 different leaves from randomly selected plants from each treatment were observed under a compound microscope at 10× magnification with a total area of 0.86 mm^2^ (Balakrishnan et al. 2024; Ayala et al. 2024).

### 2.8 Flower Count

We recorded the flowering data by counting the number of flowers per plant from each treatment (*n* = 16) for both cultivars. First flowering was observed in the sixth week after sowing. Flower counts were recorded every other day over a 10‐day period.

### 2.9 Yield Traits

For yield, pod data was collected at the pod filling stage before reaching final maturity. Then, the final pod data (after which no more new pods were formed) was recorded for both treatments for each cultivar. Once harvested, the number of two‐seeded pods, three‐seeded pods, and empty pods per plant (*n* = 15) was recorded. For seed data, the total number of seeds, seed weight (g) (100 seeds per treatment) and seed diameter (mm) was measured. A digital vernier caliper was used to measure the seed diameter of 10 randomly selected seeds per treatment.

### 2.10 Statistical Analysis

All analyses were performed in the R environment using standard statistical analysis methods using the data.table package (R Core Team 2023) Analysis of variance (ANOVA) followed by Tukey's HSD test was performed for the continuous data for germination, nitrogen, physiological, trichome density, and yield traits. Throughout the analysis, the assumptions of ANOVA were also accounted for, and log/sqrt transformation or non‐parametric Kruskal–Wallis test followed by Dunn's test was conducted whenever assumptions were not met.

## 3 Results

### 3.1 Transgenerational Effects on Germination

**FIGURE 2:**
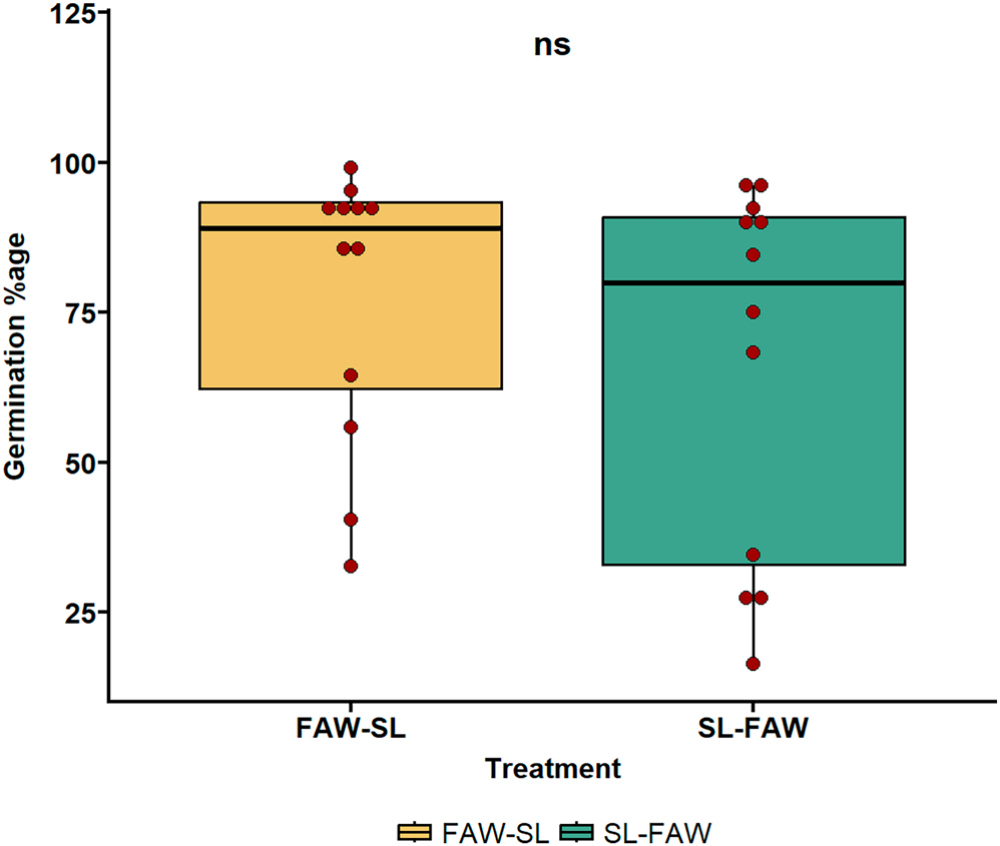
Germination percentage of transgenerational plants whose parents were under sequential herbivory of FAW and SL. FAW‐SL represents the treatment where FAW is the initial attacker and SL is the second attacker, and SL‐FAW represents the treatment where SL comes first followed by FAW. ns, not significant.

The germination percentage of the progeny seeds which were under sequential herbivory stress during the parental generation was not different across the two treatments (FAW‐SL and SL‐FAW) (Figure 2; *p* = 0.6211).

### 3.2 Effects of Parental Sequential Herbivory on Root Development

The effect of parental sequential herbivory was not observed in the progeny root traits. Progeny of plants under FAW‐SL treatment, although non‐significant, had an incremental increase in total root length (*p* = 0.403; Figure S1c), fine root length (*p* = 0.965; Figure S1d), surface area (*p* = 0.245; Figure S1d) and number of tips (*p* = 0.38; Figure S2a) compared to those under SL‐FAW treatment. Traits like the number of forks (*p* = 0.776; Figure S2b), crossings (*p* = 0.575; Figure S2c), root volume (*p* = 0.641; Figure S1a) and final root volume (*p* = 0.545; Figure S1b) were also non‐significant across the treatments.

### 3.3 Effects of Parental Sequential Herbivory on Nitrogen and Protein Percentage of Progeny Plants

**FIGURE 3:**
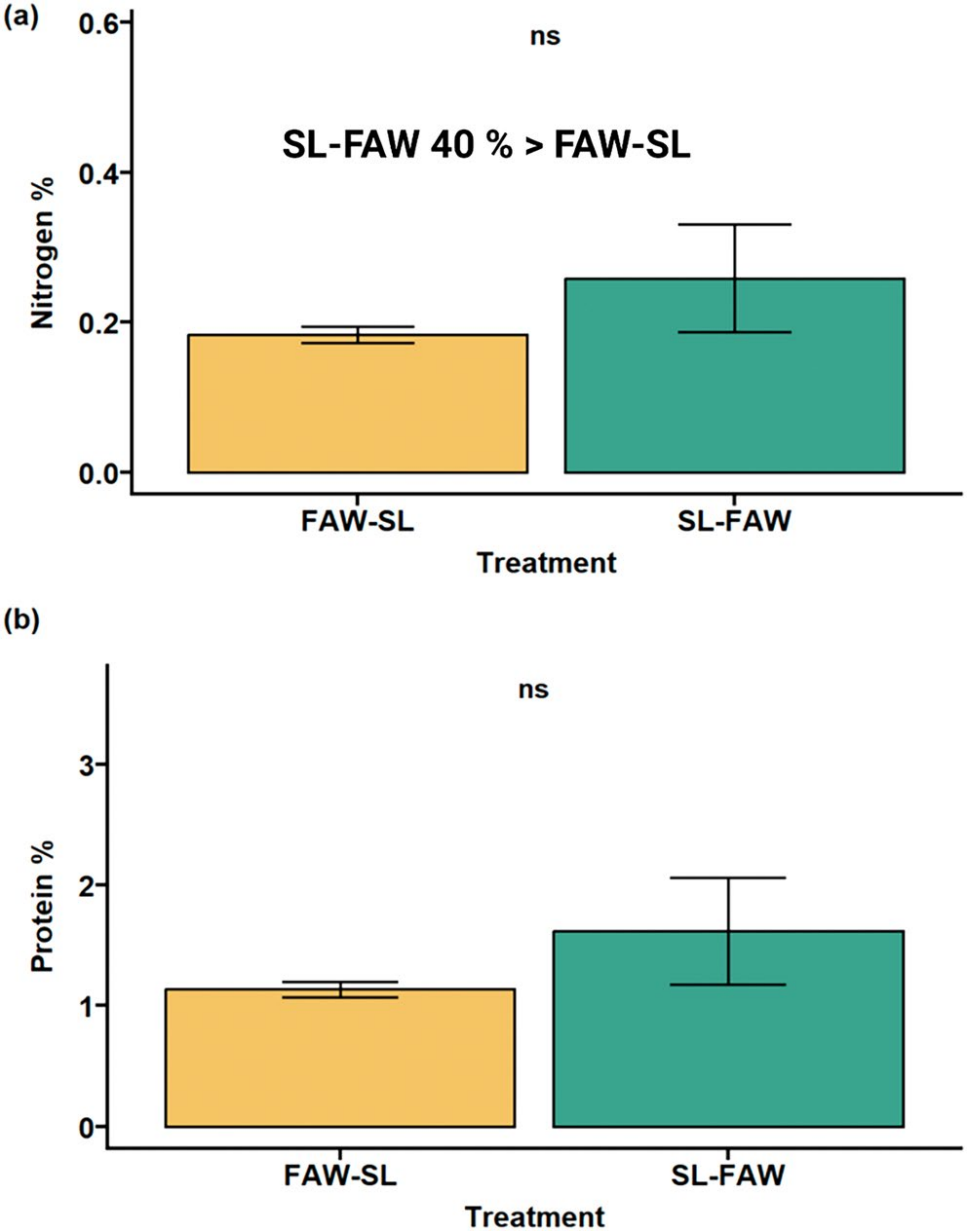
Estimation of nitrogen and protein percentage in cotyledons of transgenerational seedlings. (a) Nitrogen percentage and b) protein percentage of plants whose parents were exposed to sequential herbivory (FAW‐SL: FAW as initial attacker and SL as sequential attacker and SL‐FAW: SL followed by FAW). ns, not significant.

Results from cotyledon nitrogen and protein estimation showed that overall nitrogen percentage did not differ significantly across treatments; however, a clear trend was observed (Figure 3a,b; *p* = 0.367, *p* = 0.344). Plants under SL‐FAW treatment had around 40% higher nitrogen percentage in the transgenerational plants than the treatment FAW‐SL. A similar trend was seen in the protein percentages.

### 3.4 Transgenerational Effects on Soybean Physiological Traits

**FIGURE 4:**
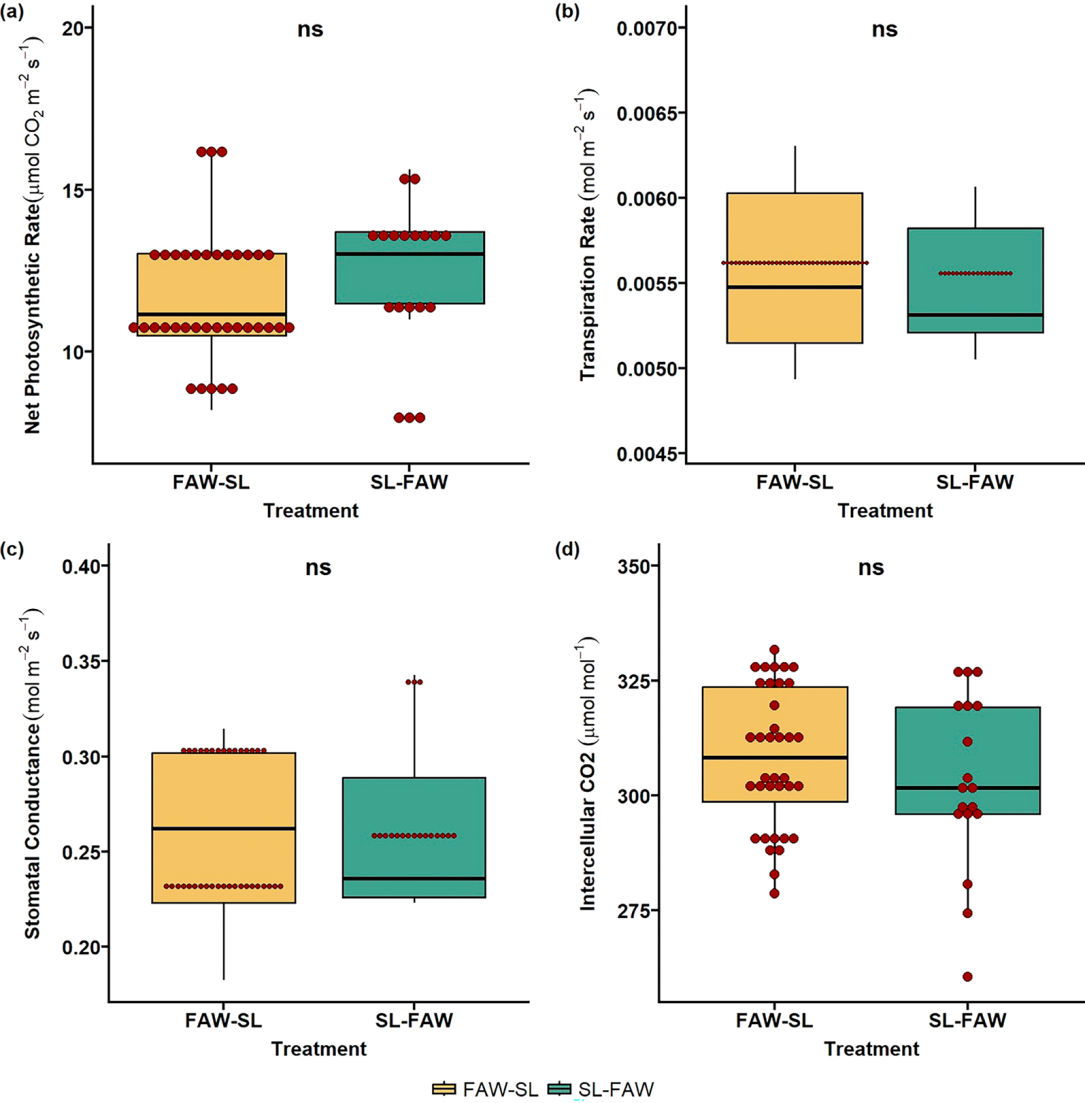
Plant physiological traits in transgenerational plants at vegetative stage (V3). Impact of parental sequential herbivore attack of fall armyworm (FAW) and soybean looper (SL) in two combinations: FAW‐SL (FAW comes first, SL as second attacker) and SL‐FAW (SL as first attacker, SL as second attacker) on progeny traits like (a) net photosynthesis, (b) transpiration rate, (c) stomatal conductance, and (d) intercellular CO_2_. ns, not significant.

**FIGURE 5:**
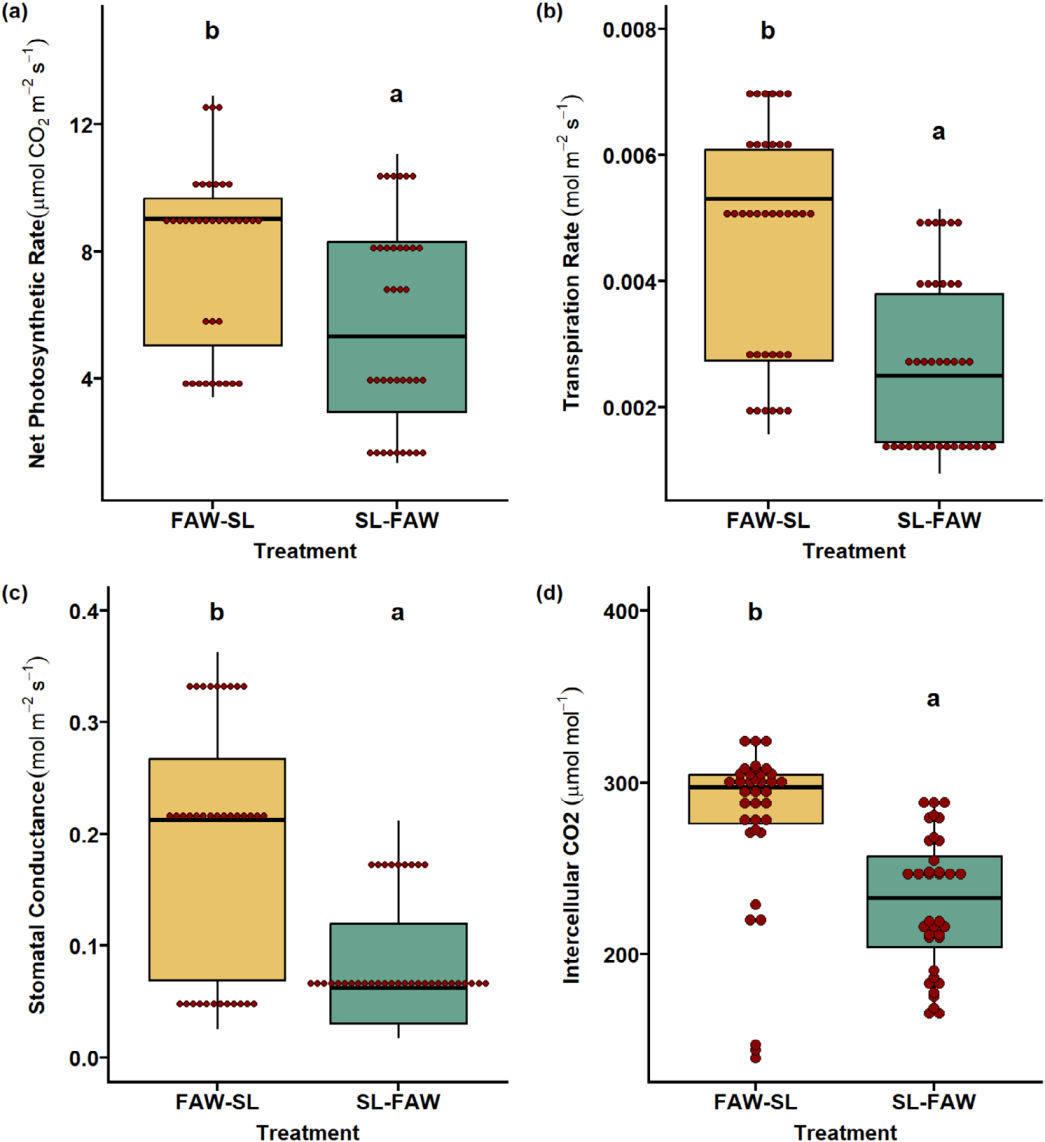
Plant physiological traits in transgenerational plants at reproductive stage (R2). Impact of parental sequential herbivore attack of fall armyworm (FAW) and soybean looper (SL) in two combinations: FAW‐SL (FAW comes first, SL as second attacker) and SL‐FAW (SL as first attacker, SL as second attacker) on progeny traits like (a) net photosynthesis, (b) transpiration rate, (c) stomatal conductance, and (d) intercellular CO_2_. Different letters in treatments indicate significant differences at the 5% level of significance.

In transgenerational plants, plant physiological traits like net photosynthesis, stomatal conductance, transpiration, and intercellular CO_2_ were recorded at two phenological stages (V3 and R2). We observed no significant changes in these traits across the two treatments in progeny plants at the V3 stage (Figure 4). In the reproductive stage (R2), an interesting pattern across all physiological traits was observed. The results revealed that progeny of plants which experienced an initial attack by FAW followed by SL (FAW‐SL) had significantly higher net photosynthesis, transpiration rate, stomatal conductance, and intercellular CO_2_ (*p* < 0.05; Figure 5).

### 3.5 Effect of Parental Sequential Herbivory on Flowering

**FIGURE 6:**
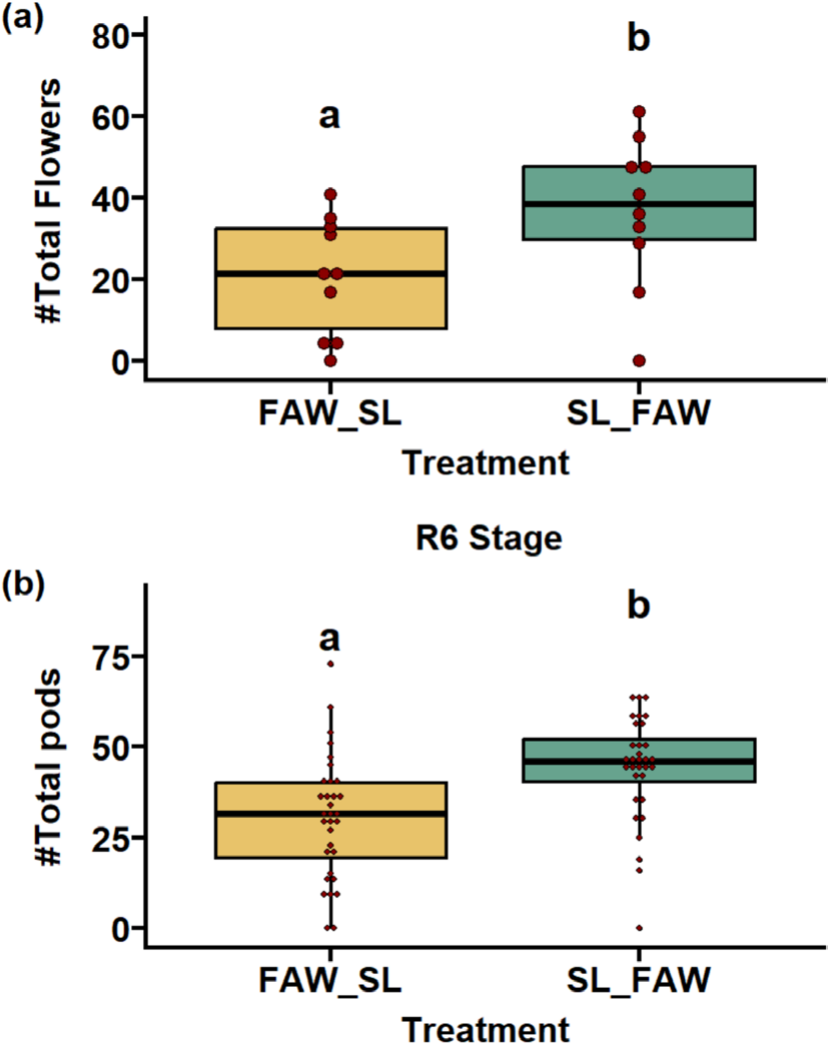
Effect of sequential herbivory of fall armyworm (FAW) and soybean looper (SL) on (a) number of flowers and (b) early pod number of progeny plants under two treatments: FAW‐SL represents the progeny of plants whose parents experienced initial attack by FAW followed by SL whereas in SL‐FAW, the attack sequence was reversed. Different letters in treatments indicate significant differences at the 5% level of significance. ns, not significant.

We examined the total number of flowers of progeny plants whose parents experienced sequential attack of FAW and SL in two sequences: FAW‐SL and SL‐FAW. We observed that the plants started flowering in Week 6 after planting. After 10 days post first day of flowering, the total number of flowers in the plants under SL‐FAW herbivory in the parental generation was significantly higher than the plants under FAW‐SL treatment (*p* < 0.05; Figure 6a).

### 3.6 Trichome Density of Transgenerational Plants at Two Phenological Stages

**FIGURE 7:**
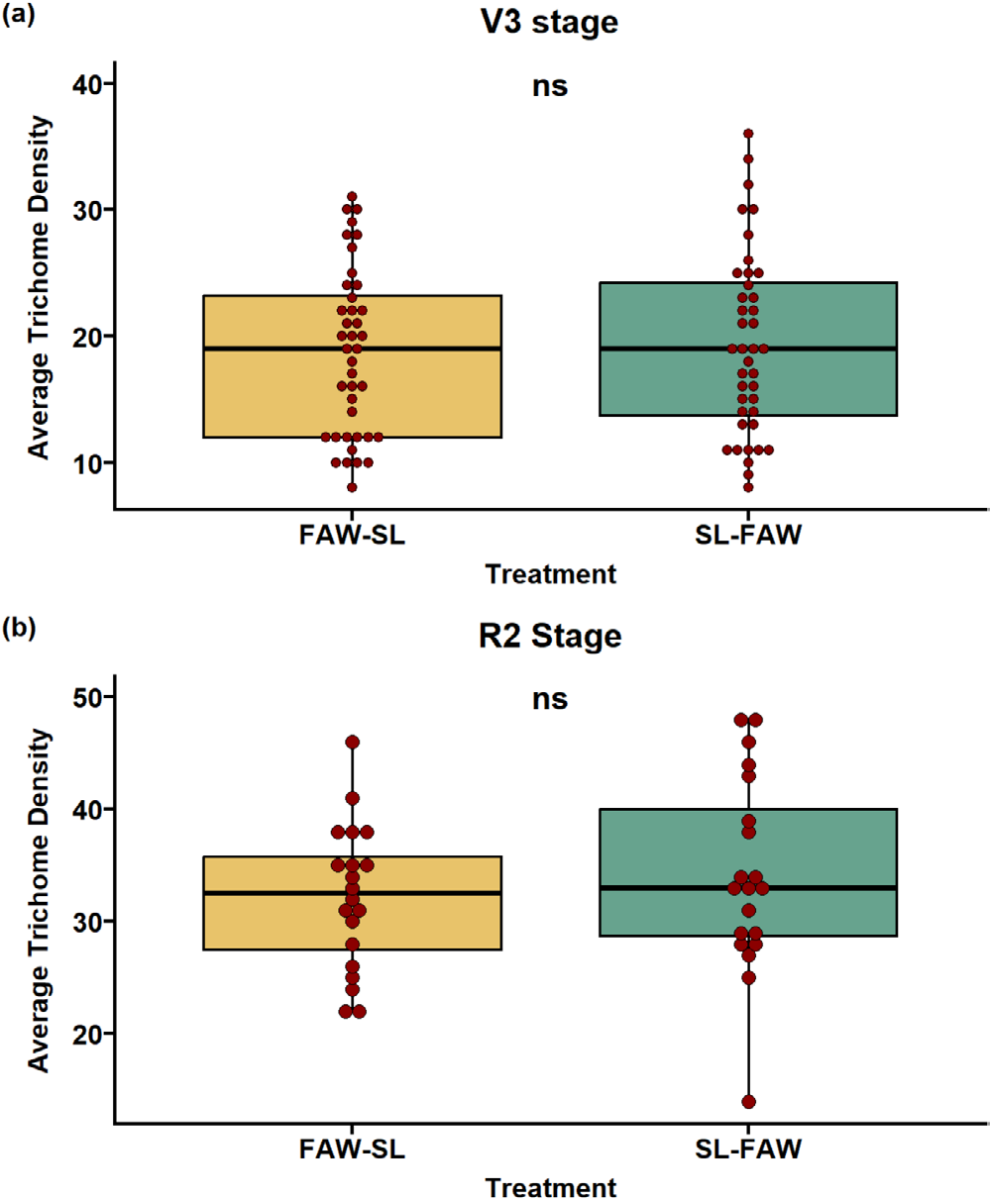
Transgenerational effects of leaf trichome density at two growth stages. Effect on (a) average leaf trichome density at V3 stage and (b) R2 stage of plants whose parents were under sequential herbivory of FAW and SL in two different sequences: FAW‐SL (FAW—initial attacker and SL—sequential attacker) and SL‐FAW (SL—initial attacker and FAW—sequential attacker). ns, not significant.

The effects of parental sequential herbivory on the leaf trichome density of progeny of two treatment combinations: FAW‐SL (FAW—first attacker, SL—second attacker) and SL‐FAW (SL—first attacker, FAW—second attacker) were recorded at two growth stages (V3 and R2). The results revealed that there were no significant differences in the average trichome density across the two treatments at both growth stages (*p* = 0.689 at V3; Figure 7a; *p* = 0.412 at R2; Figure 7b).

### 3.7 Transgenerational Impact of Sequential Herbivory on Yield and Fitness Traits

**FIGURE 8:**
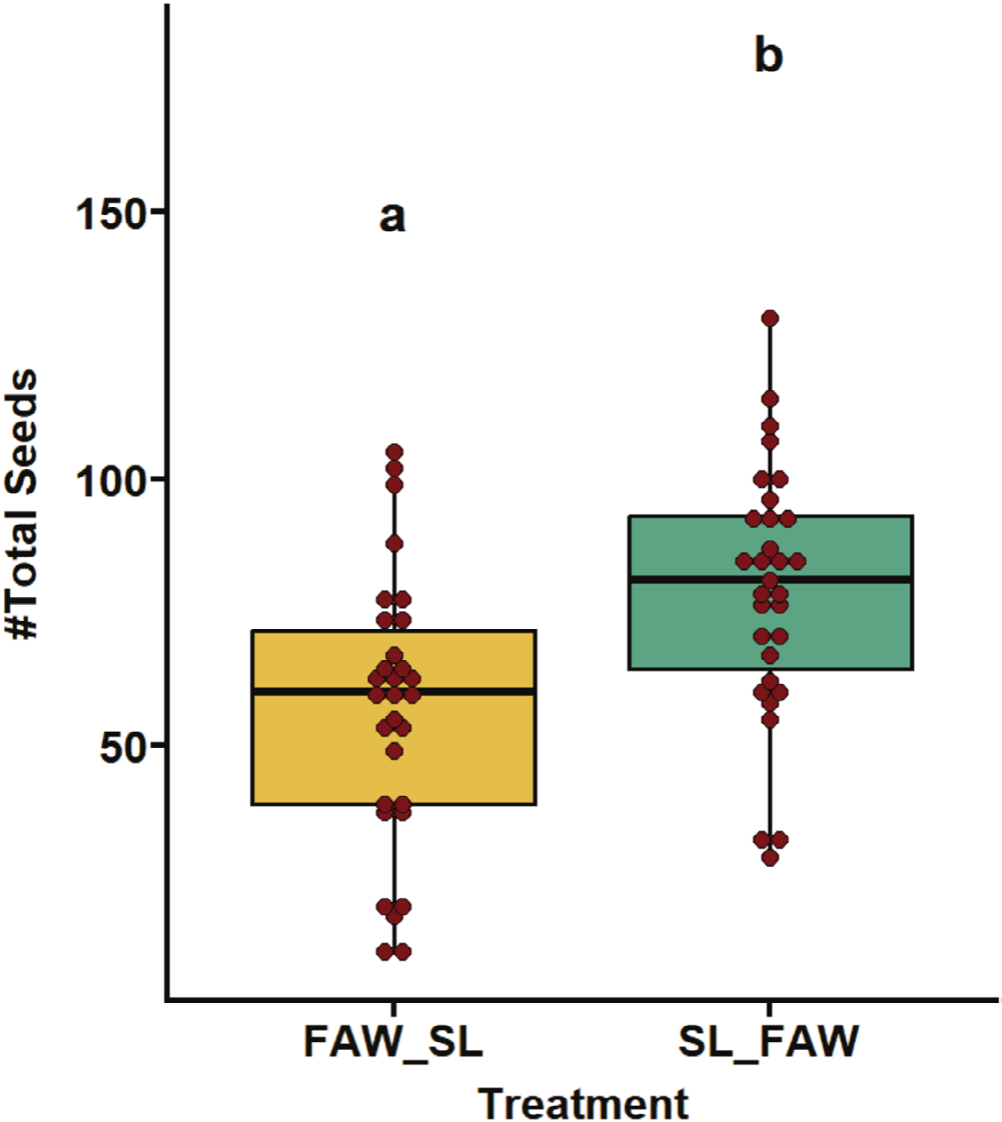
Transgenerational effects of parental sequential herbivory on total seed number across two treatments: FAW‐SL (FAW as the initial attacker and SL as second attacker in parental generation) and SL‐FAW (SL as the initial attacker and FAW as second attacker in parental generation). Different letters in treatments indicate significant differences at the 5% level of significance.

**FIGURE 9:**
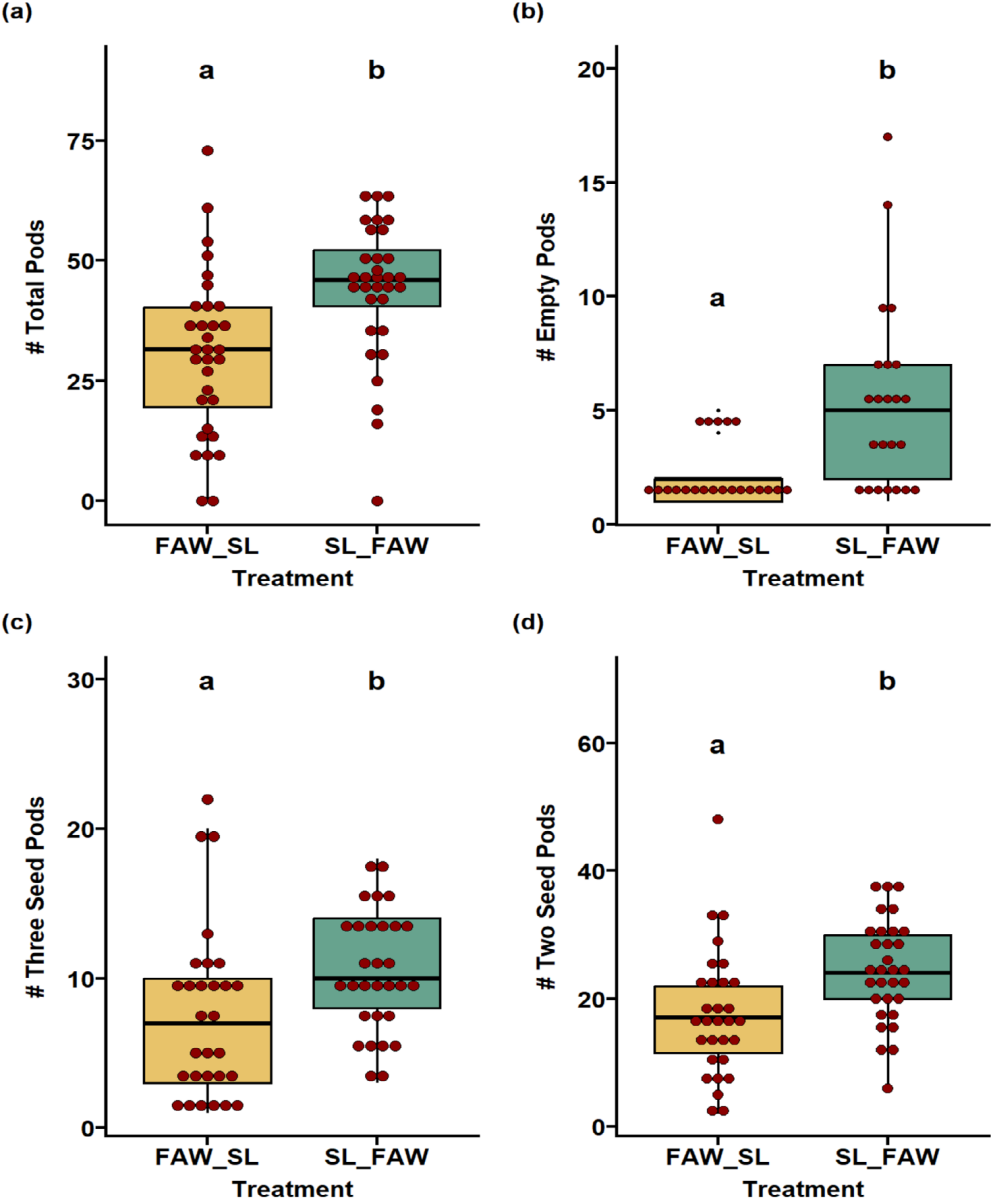
Transgenerational effects of sequential herbivory on pod traits. Effect on (a) total pod number (b) number of empty pods per plant, (c) number of three seeded pods per plant, and (d) number of two seeded pods per plant from transgenerational plants whose parents were exposed to sequential attack of FAW and SL in two sequences; (i) FAW‐SL which represents treatment where FAW as initial attacker and SL as sequential attacker and (ii) SL‐FAW treatment where sequence of attacking herbivore was reversed. Different letters in treatments indicate significant differences at the 5% level of significance.

The yield and fitness traits of progeny plants whose parents were exposed to sequential attack of FAW and SL were significantly impacted. The sequential herbivory of SL‐FAW showed significant transgenerational effects on the majority of the yield traits. We observed that the traits like early pod number (*p* < 0.05; Figure 6b), total seed number (*p* < 0.05; Figure 8), total pod number, empty pods, two‐seeded pods, and three‐seeded pods (*p* < 0.05; Figure 9) were significantly higher under SL‐FAW progeny plants than FAW‐SL progeny plants. Moreover, the weight of 100 seeds per treatment (*p* = 0.561 respectively; Figure S3b–d) and seed diameter (*p* = 0.33; Figure S3a) were not significant across the transgenerational plants from FAW‐SL and SL‐FAW treatments.

### 3.8 Cultivar Effects on Transgenerational Responses

Besides evaluating the herbivore sequence effects, we also looked at the influence of cultivars “Blackhawk and Magellan” on soybean traits (Table S1). No significant effect of cultivars was observed on the germination percentage (Figure S4a). We observed significant cultivar effects on early growth traits (nitrogen percentage, Figure S4b and root traits like tips, forks, crossings and root length, Figure S5a–d). Trichome density was also different between the two cultivars at R2 but no difference at V3 stage was observed (Figure S6). In V3 stage, “Magellan” showed significantly higher physiological response in most of the traits (Figure S7a–c); however, no difference was observed at R2 stage (Figure S8a–d). There was no significant difference between the cultivars in the reproductive traits like (pod number at R6, final pod number, number of flowers and seeds) (Figure S9a–d). Two‐ and three‐seeded pods, empty pods, seed diameter, seed weight were also different among cultivars (Figure S10). Although cultivar background contributed to variation in some traits, the main transgenerational patterns in fitness and physiology were largely consistent.

## 4 Discussion

Our study provides possibly the first evidence showcasing transgenerational effects of sequential herbivory by two chewing herbivores—FAW and SL on—soybean progeny. The sequential herbivory in the parental generation resulted in significant alterations in morpho‐physiological traits, with FAW, a generalist pest, outperforming SL, a narrow‐range pest but more dominant on soybean. The findings of our current study indicate that maternal sequential herbivory of FAW and SL in alternating sequences leaves critical transgenerational imprints on physiology and fitness traits. Particularly, the sequence in which SL is the initial attacker (SL‐FAW) resulted in enhanced transgenerational physiological and reproductive output.

Contrastingly, initial herbivory by FAW in the parental generation (FAW‐SL) dampened the transgenerational responses of the progeny physiology and fitness, echoing the concept that the pattern of attacking sequence in the maternal generation plays a critical role in shaping offspring performance. Taken together, our results show that the sequence of biotic stress in the parental generation can profoundly shape transgenerational phenotypic expression, thereby necessitating thorough investigation of how multiple, sequential stresses affect crop resilience across generations.

### 4.1 Transgenerational Impacts of Sequential Herbivory on Progeny Early Growth

Herbivore sequence attack in parental generation (FAW‐SL or SL‐FAW) did not impact early growth traits. Germination percentage of FAW‐SL and SL‐FAW progeny was similar, revealing that parental stress did not affect early germination. Previous studies have reported both positive and negative effects of herbivory on seed traits (Roach and Wulff 1987; Karban and Lowenberg 1992; Agrawal 1998, 2001; Moreira et al. 2015). For instance, herbivory by *M. sexta* resulted in positive seedling emergence in *Solanum carolinense* (Nihranz et al. 2020). Our findings run counter to the sequential herbivore study of two generalist leaf‐chewing herbivores ( *Spodoptera eridania* and *Diabrotica balteata* ) on *Phaseolus lunatus* plants, where the order of herbivore attack significantly influenced seed germination (Kellenberger et al. 2018). The possible explanation of having no detectable effect on transgenerational seed germination in our study could be explained from the viewpoint of Fenner (1991), highlighting that germination traits may be buffered against parental stress to ensure offspring viability. Instead of altering germination per se, it is likely plants may have preferentially affected post‐germination traits, consistent with trait‐specific transgenerational plasticity (Herman and Sultan 2011).

Similar to the germination pattern, transgenerational changes in root traits were not significant. Although FAW‐SL progeny exhibit slight increases in some of the root traits, these differences were not statistically significant. Herbivory‐induced transgenerational effects on the roots of an invasive plant, alligator weed, *Alternanthera philoxeroides* , were found to be transient, aligning with our observation of no significant transgenerational root traits (Fu et al. 2023).

While early growth traits remained stable, a shift in cotyledon nitrogen content (and thereby protein percentage) was observed. We found that parental sequential herbivory impacted transgenerational cotyledon nitrogen; SL‐FAW progeny had 40% higher nitrogen than FAW‐SL. Plants strategically allocate nitrogen to specific tissues to be used for defense compounds or for compensatory growth to enhance plant survival and reproductive fitness (Kafle et al. 2017). Possibly, with SL as initial attacker in parental sequential attack, maternal plants might have adopted a bet‐hedging strategy, enhancing nitrogen provisioning for early‐stage growth in the progeny (Latzel et al. 2014). Interestingly, the higher nitrogen content in SL‐FAW progeny does not seem to translate into germination or root traits. The speculative answer for this apparent “disconnect” is that the increase in nitrogen represents a form of adaptive provisioning, that lines with the concept of “latent transgenerational plasticity.” By this concept, offspring may be “primed” by parental stress with some traits not constitutively expressed unless triggered (Galloway and Etterson 2007; Herman and Sultan 2011).

### 4.2 Enhanced Physiological Response in Transgenerational Plants

A differential physiological response was observed in our parental generation, wherein we observed an increase in the physiological traits in the initial herbivory, whereas this was not evident during sequential attack. One of the interesting results from our previous experiment was that the plants under SL‐FAW herbivory exhibited higher stomatal conductance than the control plants, suggesting that priming influences the physiology of the plant. In this study, although no significant effect across all physiological traits in V3 stage was observed, FAW‐SL had consistently enhanced physiological response than SL‐FAW progeny in R2 stage. These findings suggest that initial attack by FAW may have primed stress responses in progeny (Rasmann et al. 2012). Sequential attack in the parent generation may have altered hormonal networks, resulting in enhanced physiological response in progeny (Kuśnierczyk et al. 2008; Nguyen et al. 2016). This is in line with the ecological theory that parental experience shapes progeny traits to optimize survival under future stress events (Agrawal 2001; Galloway and Etterson 2007). Enhanced physiological performance in FAW‐SL progeny may also be explained by the higher specificity and defensive elicitation potential of SL compared to the generalist, FAW (Ali and Agrawal 2012).

### 4.3 Trichome Density Remained Unaltered in the Progeny

Trichomes, the hair‐like structures, serve as the first line of physical defense of plants against herbivores (War et al. 2012; Kariyat et al. 2013). There is a substantial literature support about the induction of trichomes upon herbivore attack within a generation (Agrawal 2002; Brian Traw and Dawson 2000; Kaur and Kariyat 2020), and that parental herbivory can enhance defense responses in the progeny (Agrawal 2001, 2002; Holeski 2007; Rasmann et al. 2012). Despite the transgenerational impact on physiological traits, there was no difference in trichome density between FAW‐SL and SL‐FAW progeny at both growth stages (V3 or R2). Our results may be interpreted within the framework of optimal defense theory (ODT) (McKey 1974), suggesting that defenses are optimized in a way that plant fitness is prioritized. Here in particular, plants might have strategically favored other traits like physiology rather than investing in a costly phenomenon of trichome production. This is again proven by the concept of ODT predicting a trade‐off between constitutive versus induced defenses, suggesting preference of transgenerational physiological priming over physical defenses (Fritz and Simms 1992; Zangerl and Rutledge 1996).

### 4.4 Positive Transgenerational Effects on Soybean Fitness

A strong transgenerational response on plant fitness and reproductive output was exerted by parental sequential herbivory. A significant increase in yield traits (number of flowers, early and final pod number, two‐and three seeded pods, total seed number) was recorded in progeny derived from SL‐FAW parents compared to progeny where FAW was the initial attacking herbivore (FAW‐SL). This provides strong evidence that sequential herbivory in parents can prime offspring reproductive investment. It is well established that parental stress can prime rapid defense or reproductive response in offspring upon subsequent attack (Van Hulten et al. 2006). Our findings are consistent with the study by Steets and Ashman (2010) showing enhanced flower number in progeny of *Impatiens capensis* plants that experienced herbivory in the parental generation. Nihranz et al. (2020) reiterates this concept in their study wherein they found that previous‐generation herbivory enhanced reproductive traits in *Solanum carolinense* progeny. Potential reasons for no observable changes in seed size and weight despite higher number can be again explained through seed output maximization strategy, fitting well with bet‐hedging frameworks under uncertain environment (Latzel et al. 2014). Increase in the fitness traits in transgenerational plants under SL‐FAW group also aligns with the optimal defense theory (ODT), plants invest in the traits that maximize their fitness (McKey 1974). Here, investing in reproductive fitness rather than trichomes (costly structural defense) provides a more resource‐efficient strategy. Importantly, the differential responses in fitness traits between SL‐FAW and FAW‐SL reveal that herbivore attack sequence and identity are central drivers of transgenerational plasticity, thereby adding a layer of complexity to plant responses in multiple herbivore environments.

In conclusion, our study shows that sequential herbivory, a common and complex ecological phenomenon, triggers strong transgenerational shifts in soybean physiology and fitness traits. This study clearly supports the hypothesis that parental herbivory affects offspring success. More importantly, our study demands the mechanistic investigations to disentangle whether maternal provisioning or epigenetic changes are the key players in transgenerational responses. There is a need to move beyond studying the impact of a single herbivore on crops, given the unpredictable nature of biotic and abiotic stresses in agricultural ecosystems.

## Conflicts of Interest

The authors declare no conflicts of interest.

## Supporting information


Data S1.

## Data Availability

All raw data has been uploaded to Figshare and can be accessed under the doi https://doi.org/10.6084/m9.figshare.29386508.

## References

[pei370070-bib-0001] Adachi‐Fukunaga, S. , Y. Nakabayashi , and M. Tokuda . 2022. “Transgenerational Changes in Pod Maturation Phenology and Seed Traits of *Glycine soja* Infested by the Bean Bug *Riptortus pedestris* .” PLoS One 17, no. 3: e0263904. 35235584 10.1371/journal.pone.0263904 PMC8890626

[pei370070-bib-0002] Agrawal, A. A. 1998. “Induced Responses to Herbivory and Increased Plant Performance.” Science 279, no. 5354: 1201–1202. 9469809 10.1126/science.279.5354.1201

[pei370070-bib-0003] Agrawal, A. A. 2001. “Transgenerational Consequences of Plant Responses to Herbivory: An Adaptive Maternal Effect?” American Naturalist 157, no. 5: 555–569. 10.1086/319932 18707262

[pei370070-bib-0004] Agrawal, A. A. 2002. “Herbivory and Maternal Effects: Mechanisms and Consequences of Transgenerational Induced Plant Resistance.” Ecology 83, no. 12: 3408–3415.

[pei370070-bib-0005] Agrawal, A. A. , C. Laforsch , and R. Tollrian . 1999. “Transgenerational Induction of Defences in Animals and Plants.” Nature 401, no. 6748: 60–63.

[pei370070-bib-0006] Ali, J. G. , and A. A. Agrawal . 2012. “Specialist Versus Generalist Insect Herbivores and Plant Defense.” Trends in Plant Science 17, no. 5: 293–302. 22425020 10.1016/j.tplants.2012.02.006

[pei370070-bib-0007] Ayala, J. , A. Vasquez , D. Balakrishnan , E. Madrigal , J. George , and R. Kariyat . 2024. “Effects of Fast and Slow‐Wilting Soybean Genotypes on Fall Armyworm (*Spodoptera frugiperda*) Growth and Development.” Communicative & Integrative Biology 17, no. 1: 2354421. 38778870 10.1080/19420889.2024.2354421 PMC11110702

[pei370070-bib-0008] Balakrishnan, D. , N. Bateman , and R. R. Kariyat . 2024. “Rice Physical Defenses and Their Role Against Insect Herbivores.” Planta 259, no. 5: 110. 38565704 10.1007/s00425-024-04381-7 PMC10987372

[pei370070-bib-0009] Ballhorn, D. J. , S. Kautz , and J. M. Laumann . 2016. “Herbivore Damage Induces a Transgenerational Increase of Cyanogenesis in Wild Lima Bean ( *Phaseolus lunatus* ).” Chemoecology 26, no. 1: 1–5.

[pei370070-bib-0010] Boyko, A. , and I. Kovalchuk . 2011. “Genome Instability and Epigenetic Modification—Heritable Responses to Environmental Stress?” Current Opinion in Plant Biology 14, no. 3: 260–266. 21440490 10.1016/j.pbi.2011.03.003

[pei370070-bib-0054] Brian Traw, M. , and T. E. Dawson . 2002. “Reduced Performance of Two Specialist Herbivores (Lepidoptera: Pieridae, Coleoptera: Chrysomelidae) on New Leaves of Damaged Black Mustard Plants.” Environmental Entomology 31, no. 4: 714–722.

[pei370070-bib-0049] Bruno, L. , D. Billi , S. Bellezza , and P. Albertano . 2009. “Cytomorphological and Genetic Characterization of Troglobitic Leptolyngbya Strains Isolated from Roman Hypogea.” Applied and Environmental Microbiology 75, no. 3: 608–617. 19047394 10.1128/AEM.01183-08 PMC2632135

[pei370070-bib-0011] Colicchio, J. 2017. “Transgenerational Effects Alter Plant Defence and Resistance in Nature.” Journal of Evolutionary Biology 30, no. 4: 664–680. 28102915 10.1111/jeb.13042 PMC5382043

[pei370070-bib-0012] de Souza, L. A. , and M. F. G. Peñaflor . 2024. “Small but Strong: Herbivory by Sap‐Feeding Insect Reduces Plant Progeny Growth but Enhances Direct and Indirect Anti‐Herbivore Defenses.” Oecologia 205, no. 1: 191–201. 38782789 10.1007/s00442-024-05567-2

[pei370070-bib-0013] Dowle, M. , A. Srinivasan , J. Gorecki , et al. 2019. Package ‘data.table’. Extension of ‘data.frame’. CRAN.

[pei370070-bib-0014] Fenner, M. 1991. “The Effects of the Parent Environment on Seed Germinability.” Seed Science Research 1, no. 2: 75–84.

[pei370070-bib-0053] Fritz, R. S. , and E. L. Simms . 1992. “Ecological Genetics of Plant‐Phytophage.” In Plant Resistance to Herbivores and Pathogens: Ecology, Evolution, and Genetics, 1. University of Chicago Press.

[pei370070-bib-0016] Fu, Q. Y. , C. L. Yu , R. Dong , et al. 2023. “Transgenerational Herbivory Effects on Performance of Clonal Offspring of the Invasive Plant *Alternanthera philoxeroides* .” Plants 12, no. 5: 1180. 36904040 10.3390/plants12051180 PMC10005396

[pei370070-bib-0017] Galloway, L. F. , and J. R. Etterson . 2007. “Transgenerational Plasticity Is Adaptive in the Wild.” Science 318, no. 5853: 1134–1136. 18006745 10.1126/science.1148766

[pei370070-bib-0018] Gautam, M. , I. Shafi , and R. Kariyat . 2024. “Compensation of Physiological Traits Under Simulated Drought and Herbivory Has Functional Consequences for Fitness in Soybean ( *Glycine max* (L.) Merrill).” Environmental and Experimental Botany 226: 105944.

[pei370070-bib-0020] Herman, J. J. , and S. E. Sultan . 2011. “Adaptive Transgenerational Plasticity in Plants: Case Studies, Mechanisms, and Implications for Natural Populations.” Frontiers in Plant Science 2: 102. 22639624 10.3389/fpls.2011.00102 PMC3355592

[pei370070-bib-0021] Holeski, L. M. 2007. “Within and Between Generation Phenotypic Plasticity in Trichome Density of *Mimulus guttatus* .” Journal of Evolutionary Biology 20, no. 6: 2092–2100. 17903186 10.1111/j.1420-9101.2007.01434.x

[pei370070-bib-0022] Holeski, L. M. , G. Jander , and A. A. Agrawal . 2012. “Transgenerational Defense Induction and Epigenetic Inheritance in Plants.” Trends in Ecology & Evolution 27, no. 11: 618–626. 22940222 10.1016/j.tree.2012.07.011

[pei370070-bib-0023] Kafle, D. , A. Hänel , T. Lortzing , A. Steppuhn , and S. Wurst . 2017. “Sequential Above‐ and Belowground Herbivory Modifies Plant Responses Depending on Herbivore Identity.” BMC Ecology 17: 1–10. 28178961 10.1186/s12898-017-0115-2 PMC5299658

[pei370070-bib-0024] Karban, R. , and G. Lowenberg . 1992. “Feeding by Seed Bugs and Weevils Enhances Germination of Wild *Gossypium* Species.” Oecologia 92: 196–200. 28313051 10.1007/BF00317364

[pei370070-bib-0025] Kariyat, R. R. , C. M. Balogh , R. P. Moraski , C. M. De Moraes , M. C. Mescher , and A. G. Stephenson . 2013. “Constitutive and Herbivore‐Induced Structural Defenses Are Compromised by Inbreeding in *Solanum carolinense* (Solanaceae).” American Journal of Botany 100, no. 6: 1014–1021. 23545253 10.3732/ajb.1200612

[pei370070-bib-0026] Kaur, J. , and R. Kariyat . 2020. “Role of Trichomes in Plant Stress Biology.” In Evolutionary Ecology of Plant‐Herbivore Interaction, 15–35. Springer.

[pei370070-bib-0027] Kellenberger, R. T. , G. A. Desurmont , P. M. Schlüter , and F. P. Schiestl . 2018. “Trans‐Generational Inheritance of Herbivory‐Induced Phenotypic Changes in *Brassica rapa* .” Scientific Reports 8, no. 1: 3536. 29476119 10.1038/s41598-018-21880-2 PMC5824794

[pei370070-bib-0028] Kundu, P. , S. Grover , A. Perez , J. D. Raya Vaca , R. Kariyat , and J. Louis . 2023. “Sorghum Defense Responses to Sequential Attack by Insect Herbivores of Different Feeding Guilds.” Planta 258, no. 2: 35. 37389680 10.1007/s00425-023-04195-z

[pei370070-bib-0029] Kuśnierczyk, A. , P. E. R. Winge , T. S. Jørstad , J. Troczyńska , J. T. Rossiter , and A. M. Bones . 2008. “Towards Global Understanding of Plant Defence Against Aphids: Timing and Dynamics of Early *Arabidopsis* Defence Responses to Cabbage Aphid (*Brevicoryne brassicae*) Attack.” Plant, Cell & Environment 31, no. 8: 1097–1115. 10.1111/j.1365-3040.2008.01823.x 18433442

[pei370070-bib-0051] Latzel, V. , Š. Janeček , J. Doležal , J. Klimešová , and O. Bossdorf . 2014. “Adaptive Transgenerational Plasticity in the Perennial *Plantago lanceolata* .” Oikos 123, no. 1: 41–46.

[pei370070-bib-0031] McKey, D. 1974. “Adaptive Patterns in Alkaloid Physiology.” American Naturalist 108, no. 961: 305–320.

[pei370070-bib-0032] Moreira, X. , L. Abdala‐Roberts , J. Hernández‐Cumplido , M. A. Cuny , G. Glauser , and B. Benrey . 2015. “Specificity of Induced Defenses, Growth, and Reproduction in Lima Bean (*Phaseolus lunatus*) in Response to Multispecies Herbivory.” American Journal of Botany 102, no. 8: 1300–1308. 26290553 10.3732/ajb.1500255

[pei370070-bib-0033] Nagoshi, R. N. , E. A. Malo , S. Cruz‐Esteban , N. M. Rosas‐García , V. Herrera‐Mayorga , and R. L. Meagher . 2025. “Assessing the Potential for Fall Armyworm Exchanges Between the Two American Continents Across the Mexico‐Central America Land Bridge.” PLoS One 20, no. 3: e0308501. 40029859 10.1371/journal.pone.0308501 PMC11875382

[pei370070-bib-0034] Nguyen, D. , I. Rieu , C. Mariani , and N. M. van Dam . 2016. “How Plants Handle Multiple Stresses: Hormonal Interactions Underlying Responses to Abiotic Stress and Insect Herbivory.” Plant Molecular Biology 91: 727–740. 27095445 10.1007/s11103-016-0481-8 PMC4932144

[pei370070-bib-0035] Nihranz, C. T. , A. M. Helms , J. F. Tooker , M. C. Mescher , C. M. De Moraes , and A. G. Stephenson . 2022. “Adverse Effects of Inbreeding on the Transgenerational Expression of Herbivore‐Induced Defense Traits in *Solanum carolinense* .” PLoS One 17, no. 10: e0274920. 36282832 10.1371/journal.pone.0274920 PMC9595541

[pei370070-bib-0036] Nihranz, C. T. , W. S. Walker , S. J. Brown , M. C. Mescher , C. M. De Moraes , and A. G. Stephenson . 2020. “Transgenerational Impacts of Herbivory and Inbreeding on Reproductive Output in *Solanum carolinense* .” American Journal of Botany 107, no. 2: 286–297. 31944272 10.1002/ajb2.1402 PMC7064912

[pei370070-bib-0037] Puijalon, S. , J. P. Léna , N. Rivière , J. Y. Champagne , J. C. Rostan , and G. Bornette . 2008. “Phenotypic Plasticity in Response to Mechanical Stress: Hydrodynamic Performance and Fitness of Four Aquatic Plant Species.” New Phytologist 177, no. 4: 907–917. 18275493 10.1111/j.1469-8137.2007.02314.x

[pei370070-bib-0050] R Core Team . 2023. R: A Language and Environment for Statistical Computing. R Foundation for Statistical Computing. https://www.r‐project.org/.Rao.

[pei370070-bib-0038] Rapp, R. A. , and J. F. Wendel . 2005. “Epigenetics and Plant Evolution.” New Phytologist 168, no. 1: 81–91. 16159323 10.1111/j.1469-8137.2005.01491.x

[pei370070-bib-0039] Rasmann, S. , M. De Vos , C. L. Casteel , et al. 2012. “Herbivory in the Previous Generation Primes Plants for Enhanced Insect Resistance.” Plant Physiology 158, no. 2: 854–863. 22209873 10.1104/pp.111.187831 PMC3271773

[pei370070-bib-0040] Richards, E. J. 2011. “Natural Epigenetic Variation in Plant Species: A View From the Field.” Current Opinion in Plant Biology 14, no. 2: 204–209. 21478048 10.1016/j.pbi.2011.03.009

[pei370070-bib-0041] Roach, D. A. , and R. D. Wulff . 1987. “Maternal Effects in Plants.” Annual Review of Ecology and Systematics 18: 209–235.

[pei370070-bib-0048] Souza, B. H. , E. N. Costa , Z. A. Ribeiro , et al. 2021. “Soybean Leaf Age and Plant Stage Influence Expression of Resistance to Velvetbean Caterpillar and Fall Armyworm.” Chemoecology 31, no. 6: 377–390.

[pei370070-bib-0042] Steets, J. A. , and T. L. Ashman . 2010. “Maternal Effects of Herbivory in *Impatiens capensis* .” International Journal of Plant Sciences 171, no. 5: 509–518.

[pei370070-bib-0043] Tariel, J. , S. Plénet , and É. Luquet . 2020. “Transgenerational Plasticity of Inducible Defences: Combined Effects of Grand‐Parental, Parental and Current Environments.” Ecology and Evolution 10, no. 5: 2367–2376. 32184987 10.1002/ece3.6046 PMC7069331

[pei370070-bib-0044] Tripathi, D. K. , P. Rai , G. Guerriero , S. Sharma , F. J. Corpas , and V. P. Singh . 2021. “Silicon Induces Adventitious Root Formation in Rice Under Arsenate Stress With Involvement of Nitric Oxide and Indole‐3‐Acetic Acid.” Journal of Experimental Botany 72, no. 12: 4457–4471. 33095869 10.1093/jxb/eraa488

[pei370070-bib-0045] University of Arkansas System Division of Agriculture . 2024. “Nutrition Services—Central Analytical Laboratory.” https://cal.uada.edu/nutrition‐services/.

[pei370070-bib-0052] Van Hulten, M. , M. Pelser , L. C. Van Loon , C. M. Pieterse , and J. Ton . 2006. “Costs and Benefits of Priming for Defense in Arabidopsis.” Proceedings of the National Academy of Sciences of the United States of America 103, no. 14: 5602–5607. 16565218 10.1073/pnas.0510213103 PMC1459400

[pei370070-bib-0046] War, A. R. , M. G. Paulraj , T. Ahmad , et al. 2012. “Mechanisms of Plant Defense Against Insect Herbivores.” Plant Signaling & Behavior 7, no. 10: 1306–1320. 22895106 10.4161/psb.21663 PMC3493419

[pei370070-bib-0047] Zangerl, A. R. , and C. E. Rutledge . 1996. “The Probability of Attack and Patterns of Constitutive and Induced Defense: A Test of Optimal Defense Theory.” American Naturalist 147, no. 4: 599–608.

