# Supplementary material for: Transgenerational Imprints of Sequential Herbivory on Soybean Physiology and Fitness Traits
Source: Plant Environ Interact. 2025 Jul 4;6(4):e70070. doi: 10.1002/pei3.70070 (PMC12231198; doi:10.1002/pei3.70070)
Supplement: Supplementary file 1 — Data S1. [file PEI3-6-e70070-s001.docx]

**Table S 1: Cultivar effects on transgenerational traits**

| **Trait Analyzed** | **F Value** | $\boldsymbol{\chi}\boldsymbol{2}$ **Value** | **P Value** | **Significance** |
| --- | --- | --- | --- | --- |
| Germination Percentage | 0.015 | - | 0.899 | NS |
| Cotyledon Nitrogen % | - | 16.22 | <0.05 | Yes |
| Root Length | 0.14 | - | <0.001 | Yes |
| # Tips | - | 8.74 | 0.003 | Yes |
| # Forks | 0.59 | - | <0.001 | Yes |
| # Crossings | 0.27 | - | <0.001 | Yes |
| Trichome Density (V3) | - | 5.57 | 0.018 | Yes |
| Trichome Density (R2) | 0.22 | - | 0.447 | NS |
| Photosynthetic Rate (V3) | - | 4.87 | 0.027 | Yes |
| Stomatal Conductance (V3) | 0.28 | - | 0.047 | Yes |
| Transpiration Rate (V3) | - | 19.94 | <0.001 | Yes |
| Intercellular CO_2_  (V3) | 0.95 | - | 0.32 | NS |
| Photosynthetic Rate (R2) | 0.34 | - | 0.55 | NS |
| Transpiration Rate (R2) | 0.22 | - | 0.36 | NS |
| Intercellular CO_2_  (R2) | - | 0.0064 | 0.93 | NS |
| Stomatal Conductance (R2) | - | 1.14 | 0.28 | NS |
| Flower Number | 0.59 | - | 0.094 | NS |
| Pod Number | 0.842 | - | 0.101 | NS |
| One-Seed Pod | 0.084 | - | 0.77 | NS |
| Two-Seed Pod | - | 20.033 | <0.001 | Yes |
| Three-Seed Pod | - | 7.309 | 0.006 | Yes |
| Empty Pod | - | 9.44 | 0.002 | Yes |
| Seed Number | 0.28 | - | 0.06 | NS |
| Seed Diameter (mm) | 0.86 | - | <0.001 | Yes |
| Seed Weight (100 seeds) | 0.19 | - | <0.001 | Yes |

**Supplementary figures**

**
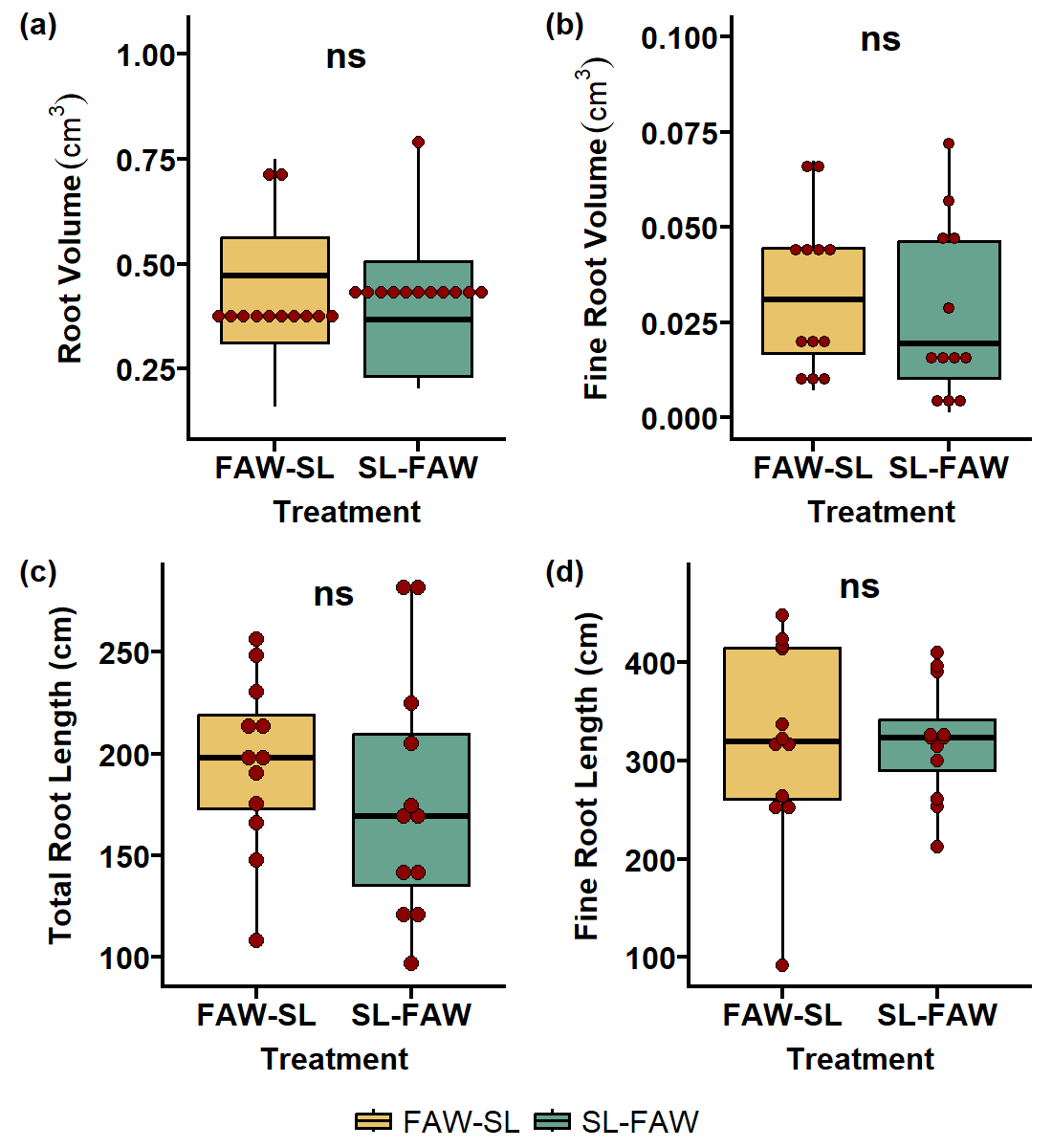
**

Figure S1: Effects of parental sequential herbivory (FAW-SL and SL-FAW) on transgenerational plants. Traits a) root volume b) fine root volume c) total root length and d) fine root length were observed in root traits. Different letters in treatments indicate significant differences at the 5 % level of significance.

**
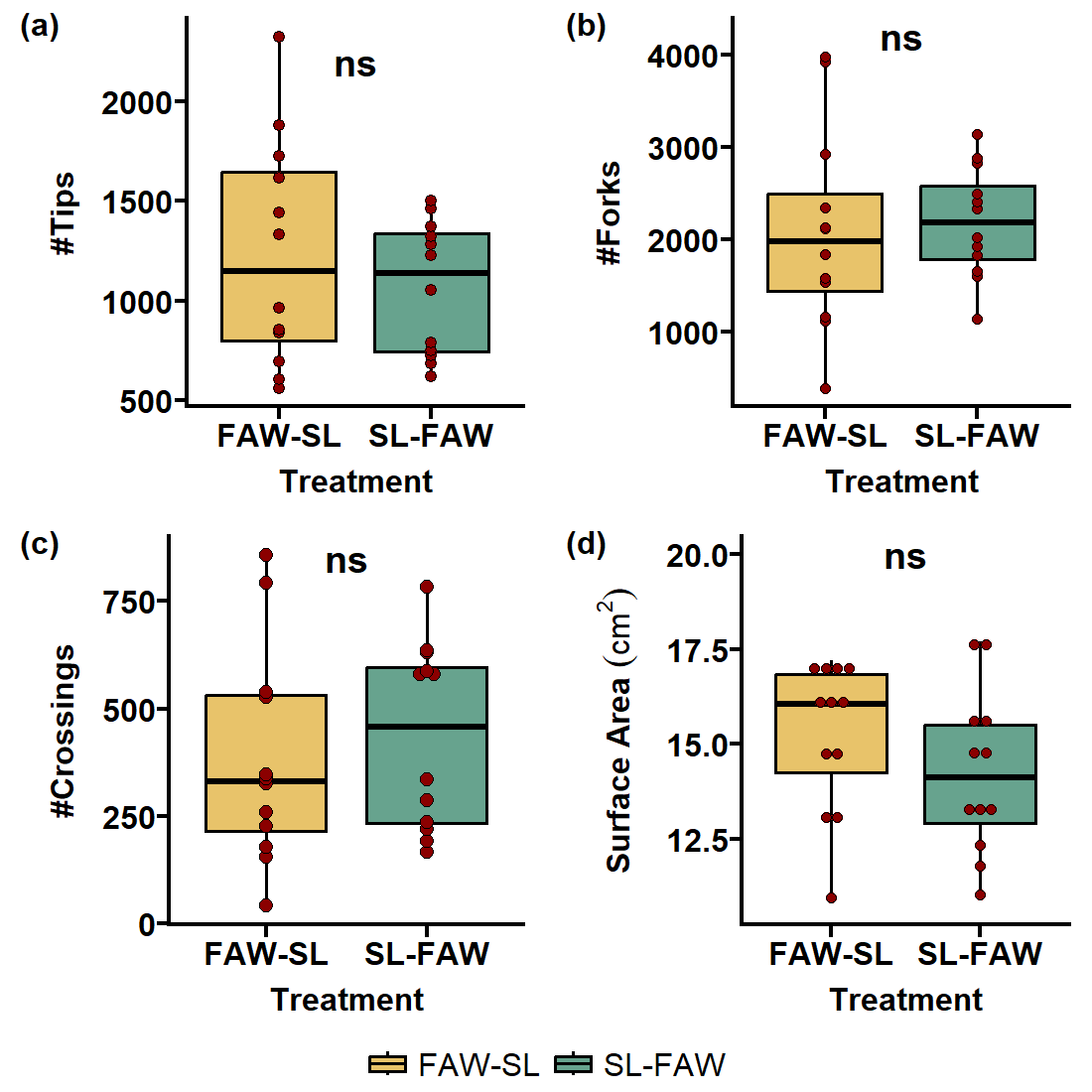
**

Figure S 2: Comparison of root morphological traits in transgenerational plants. Effect of parental sequential herbivory of FAW and SL on a) number of tips, b) number of forks, c) number of crossings, and d) surface area of transgenerational plants. Different letters in treatments indicate significant differences at the 5 % level of significance


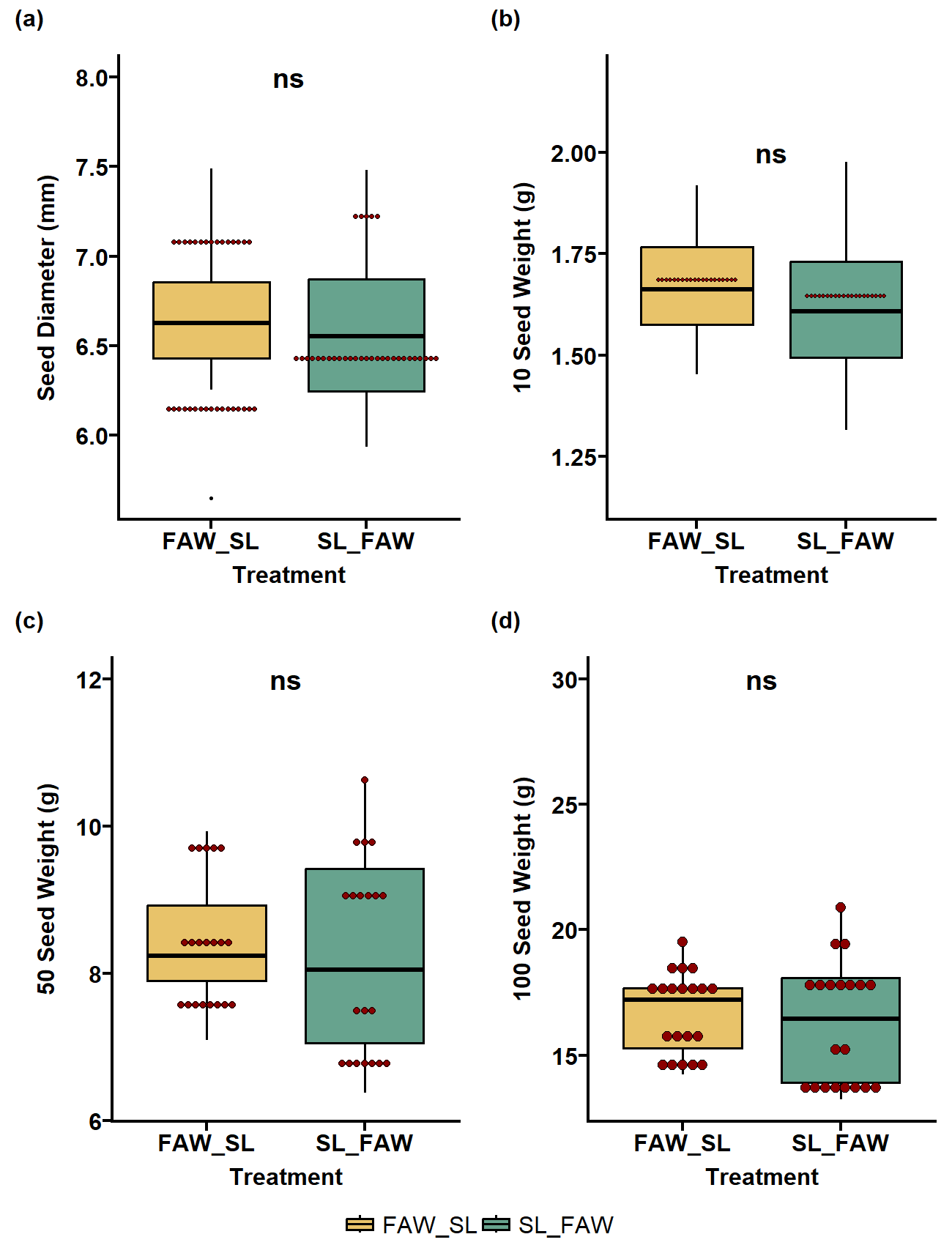


Figure S 3: Transgenerational effects of sequential herbivory on seed traits. Effect on a) seed diameter b) 10 seed weight c) 50 seed weight and d) 100 seed weight per treatment from transgenerational plants whose parents were exposed to sequential attack of FAW and SL in two sequences; i) FAW-SL which represents treatment where FAW as initial attacker and SL as sequential attacker and ii) SL-FAW treatment where sequence of attacking herbivore was reversed. Different letters in treatments indicate significant differences at the 5 % level of significance.


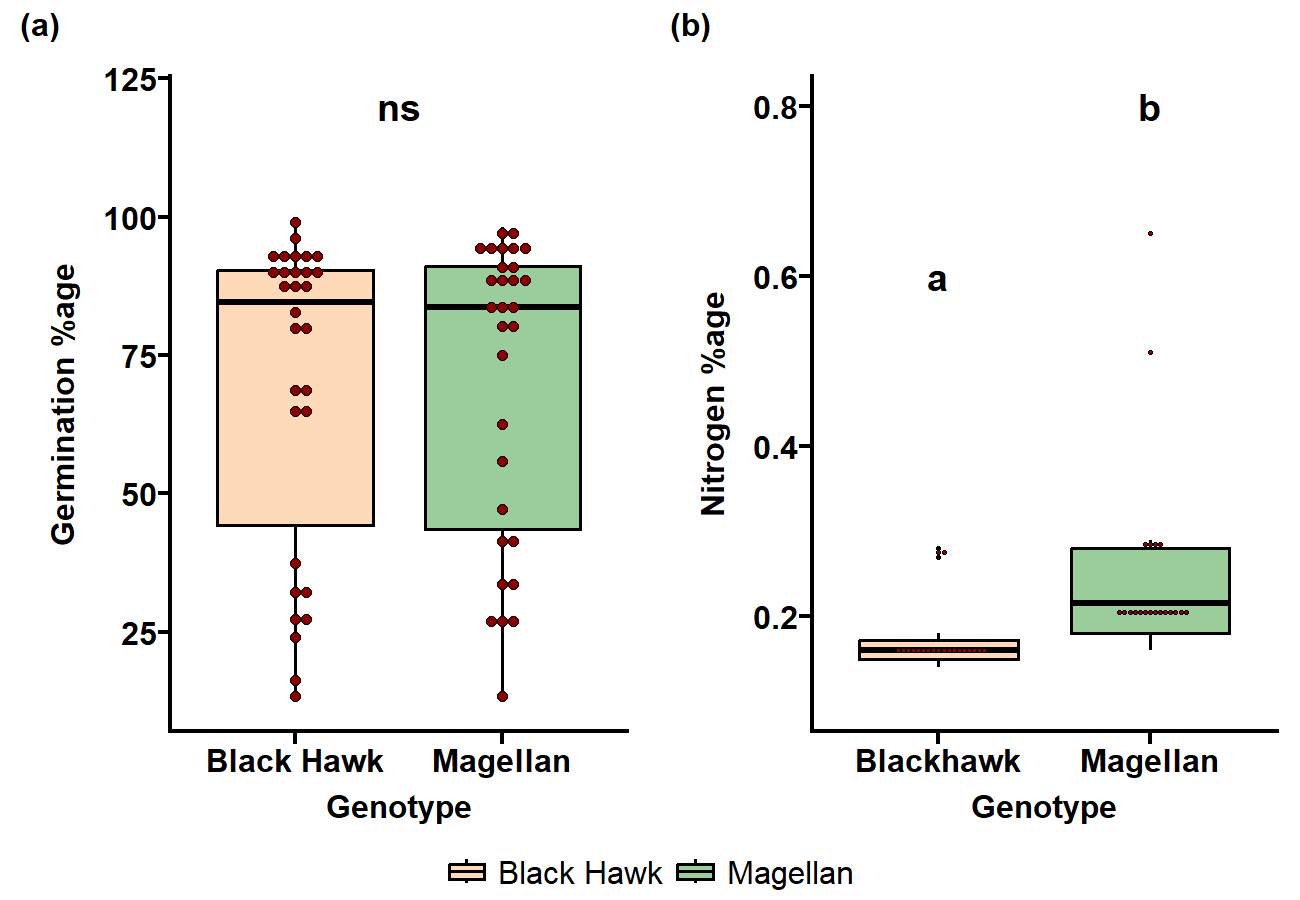


Figure S 4: Transgenerational Effects on germination percentage and nitrogen percentage across cultivars. Effect on a) germination percentage and b) 10 nitrogen percentage w.r.t cultivar “Blackhawk and “Magellan”. Different letters in treatments indicate significant differences at the 5 % level of significance.


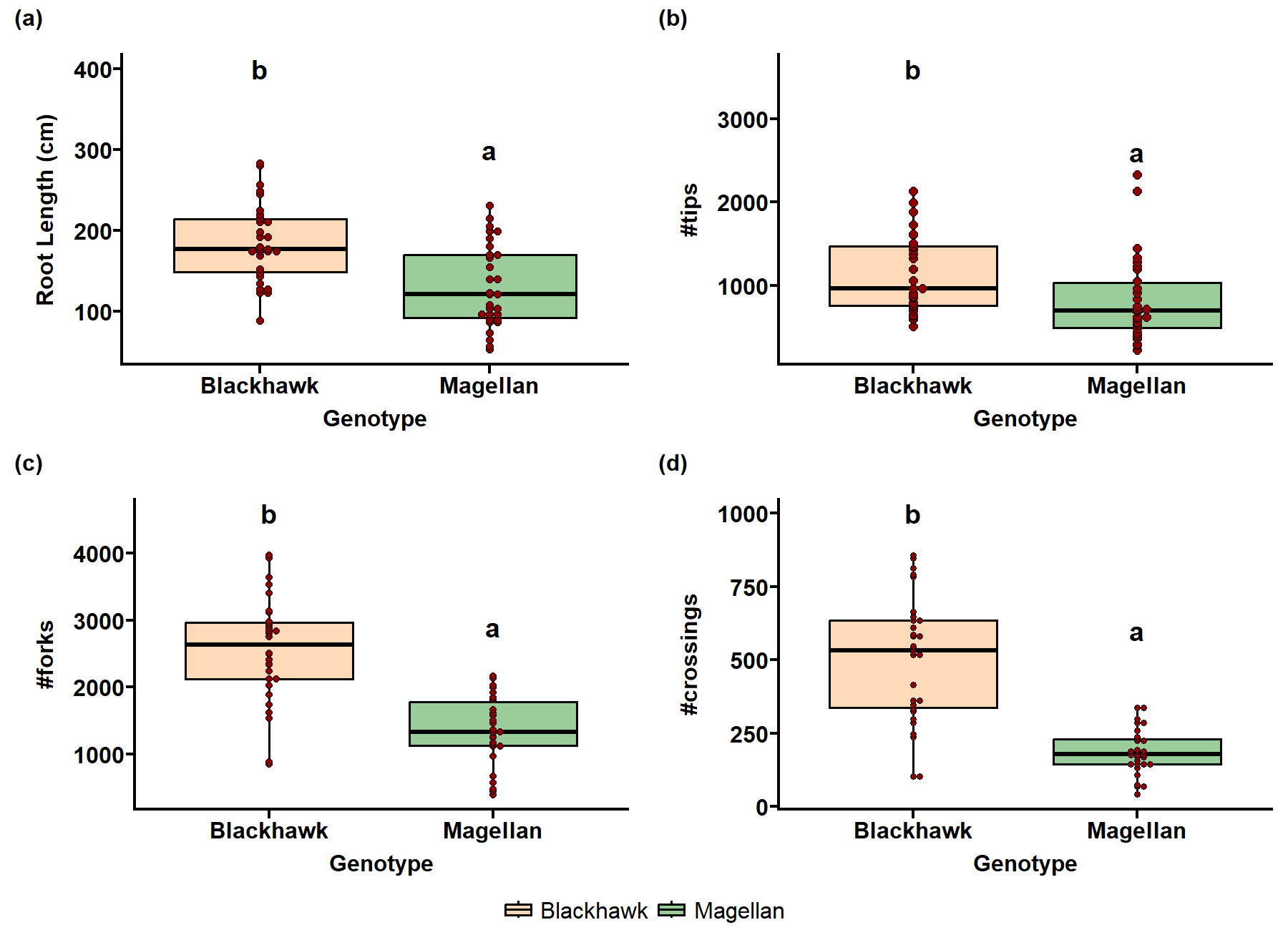


Figure S 5: Transgenerational Effects on Root Morphology. Effect on a) root length (cm) b) number of tips, c) forks, and d) crossings w.r.t cultivars “Blackhawk and “Magellan”. Different letters in treatments indicate significant differences at the 5 % level of significance.

**
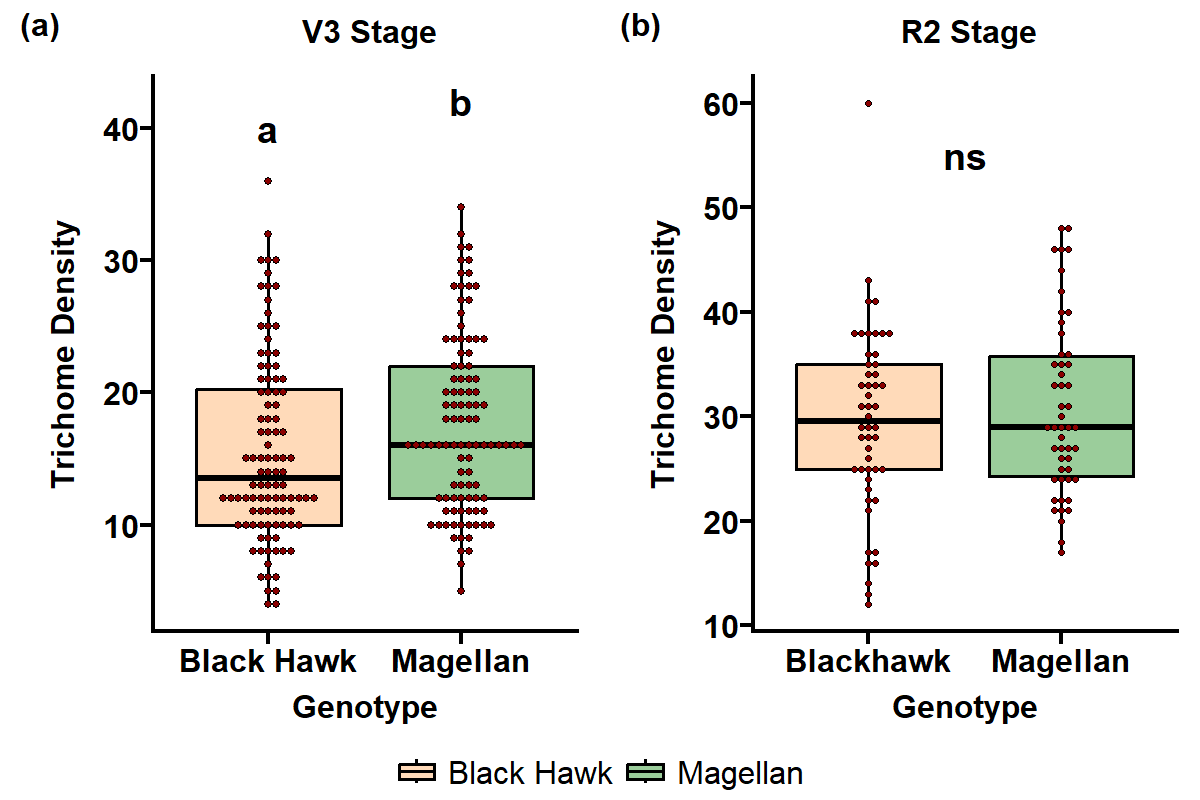
**

Figure S 6: Transgenerational Effects on Trichome Density at Two Phenological Stages. Effect on trichome density at a) V3 stage and b) R2 stage w.r.t cultivars “Blackhawk and “Magellan”. Different letters in treatments indicate significant differences at the 5 % level of significance.

**
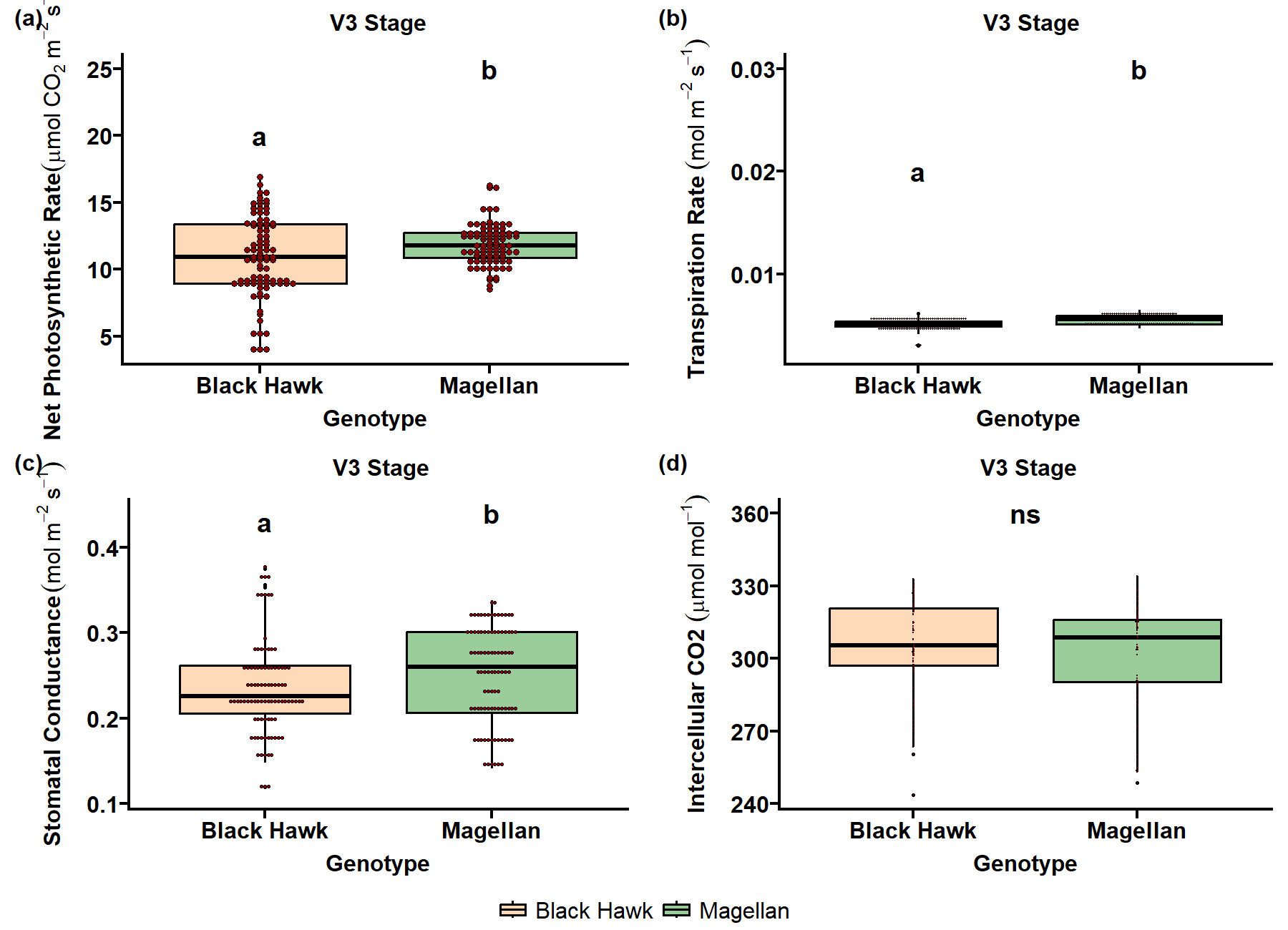
**

Figure S7: Transgenerational Effects on Plant physiology at V3 Stage. Effect on a) net photosynthesis, b) transpiration rate, c) stomatal conductance, and d) intercellular CO_2._ w.r.t cultivars “Blackhawk and “Magellan”. Different letters in treatments indicate significant differences at the 5 % level of significance.

**
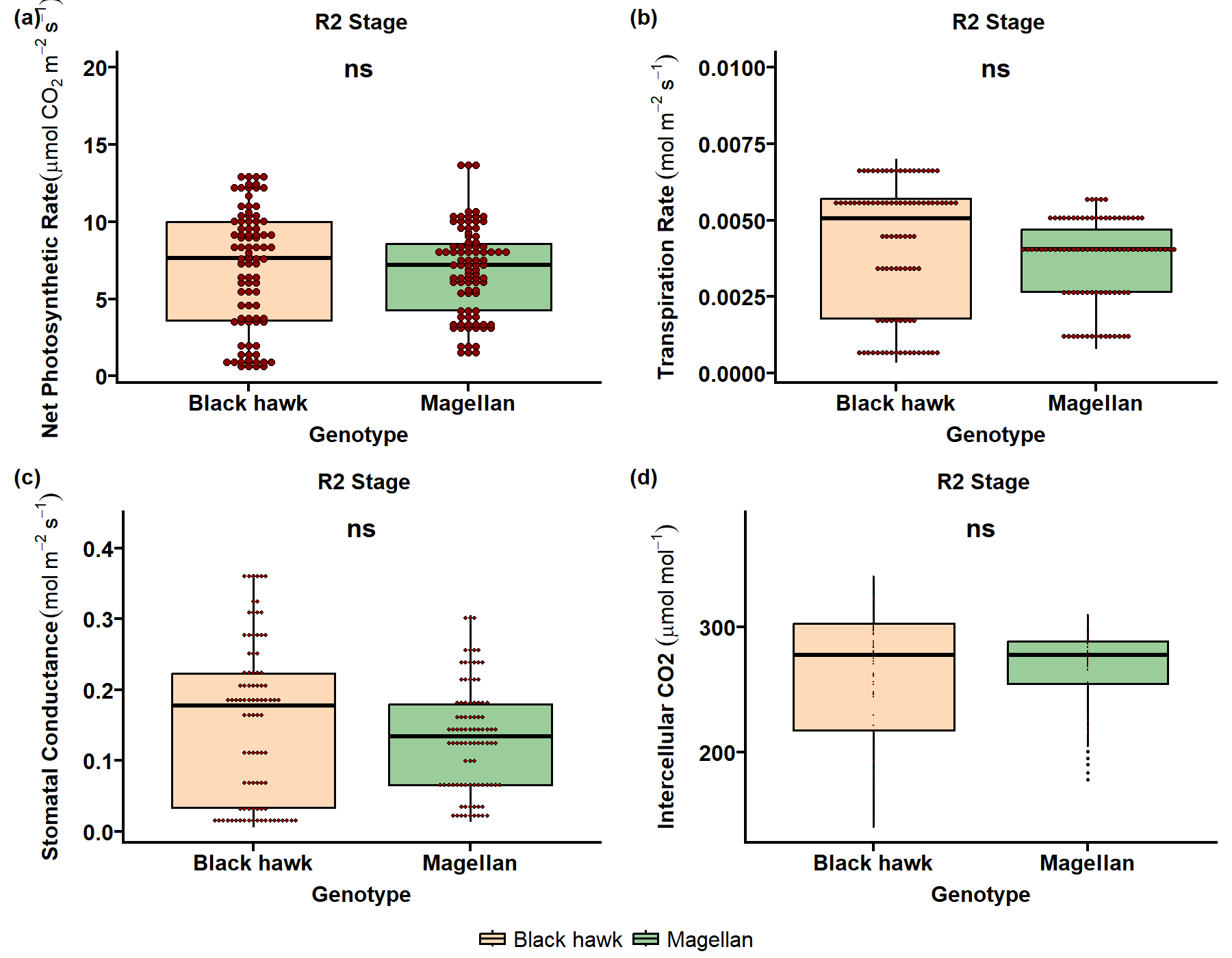
**

Figure S8: Transgenerational Effects on Plant physiology at R2 Stage. Effect on a) net photosynthesis, b) transpiration rate, c) stomatal conductance, and d) intercellular CO_2._ w.r.t cultivars “Blackhawk and “Magellan”. Different letters in treatments indicate significant differences at the 5 % level of significance.

**
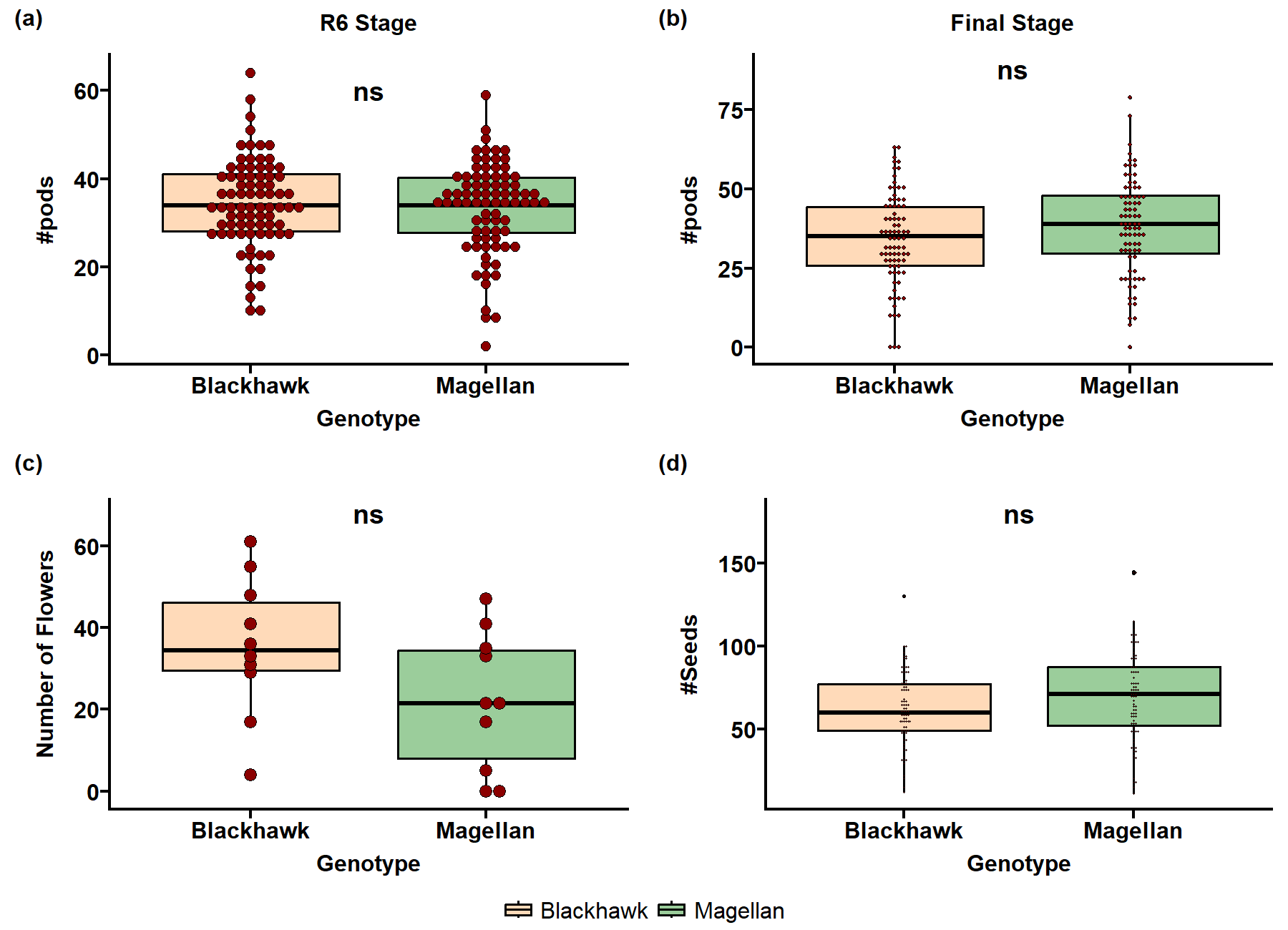
**

Figure S 9: Transgenerational Effects on Fitness Traits. Effect on a) pod number at R6 stage, b) final pod number, c) number of flowers and d) number of seeds w.r.t cultivars “Blackhawk and “Magellan”. Different letters in treatments indicate significant differences at the 5 % level of significance.


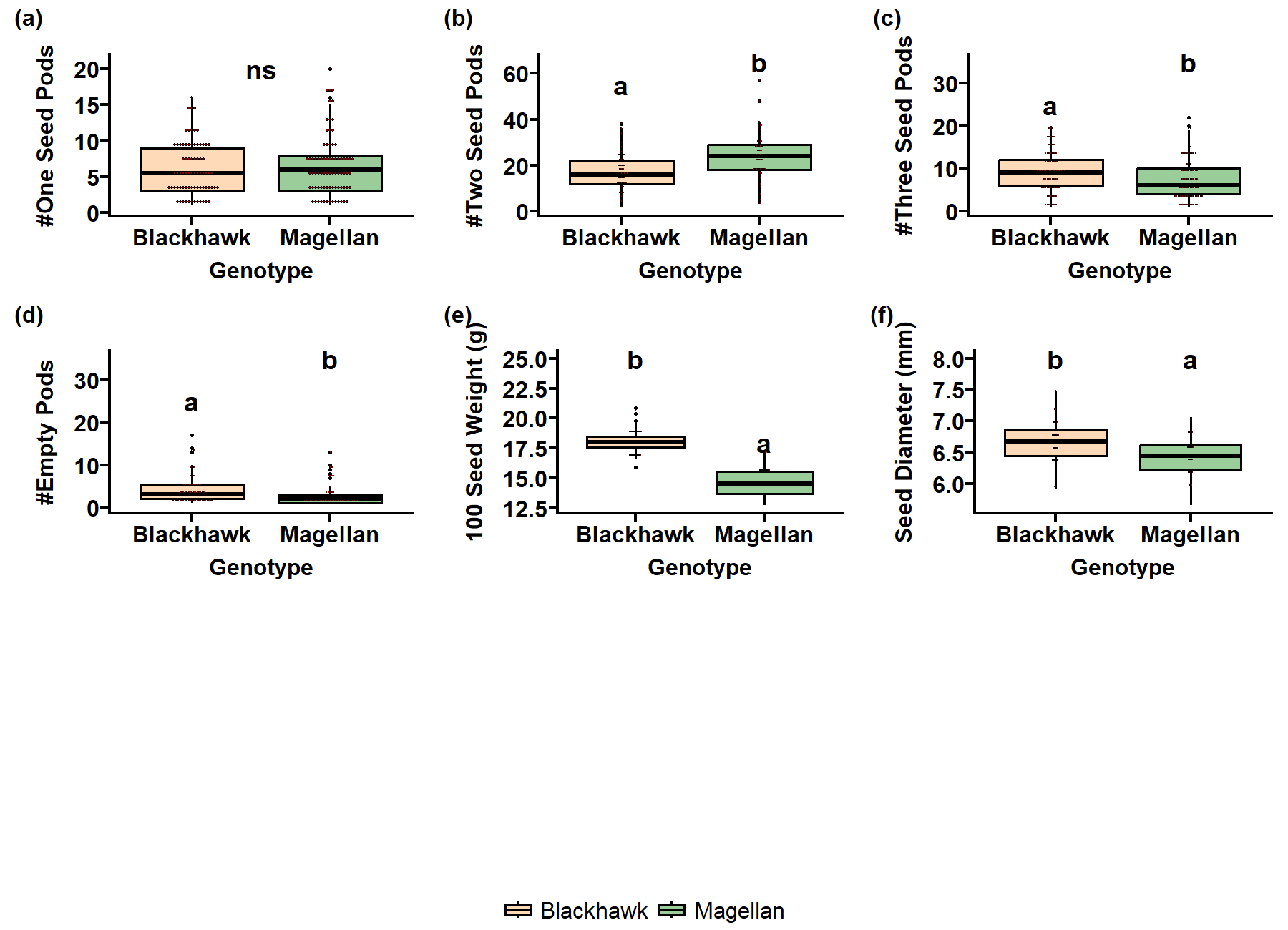


Figure S 10: Transgenerational effects on a) number of one seed pods, b) number of two seed pods, c) number of three seed pods, d) number of empty pods, e) 100 seed weight and f) seed diameter w.r.t cultivars “Blackhawk and “Magellan”. Different letters in treatments indicate significant differences at the 5 % level of significance.
